# A pair of *long intergenic non-coding RNA LINC00887* variants act antagonistically to control *Carbonic Anhydrase IX* transcription upon hypoxia in tongue squamous carcinoma progression

**DOI:** 10.1186/s12915-021-01112-2

**Published:** 2021-09-07

**Authors:** Tao Shen, Wangxiao Xia, Sainan Min, Zixuan Yang, Lehua Cheng, Wei Wang, Qianxi Zhan, Fanghong Shao, Xuehan Zhang, Zhiyu Wang, Yan Zhang, Guodong Shen, Huafeng Zhang, Li-Ling Wu, Guang-Yan Yu, Qing-Peng Kong, Xiangting Wang

**Affiliations:** 1 Department of Geriatrics, Gerontology Institute of Anhui Province, The First Affiliated Hospital, Division of Life Sciences and Medicine, University of Science and Technology of China, Hefei, China; 2 Anhui Provincial Key Laboratory of Tumor Immunotherapy and Nutrition Therapy, Hefei, China; 3 Hefei National Laboratory for Physical Sciences at the Microscale, University of Science and Technology of China, Hefei, China; 4 State Key Laboratory of Genetic Resources and Evolution/Key Laboratory of Healthy Aging Research of Yunnan Province, Kunming Institute of Zoology, The Chinese Academy of Sciences, Kunming, 650223 China; 5 Department of Oral and Maxillofacial Surgery, Peking University School and Hospital of Stomatology, Beijing, 100081 China; 6 Department of Medical Oncology, Affiliated Hospital of Hebei University, Baoding, 071000 China; 7 School of Health Services Management, Anhui Medical University, Hefei, 230032 Anhui China; 8 Department of Physiology and Pathophysiology, Peking University Health Science Center, Key Laboratory of Molecular Cardiovascular Sciences, Ministry of Education, and Beijing Key Laboratory of Cardiovascular Receptors Research, Beijing, 100191 China; 9 Center for Excellence in Animal Evolution and Genetics, Chinese Academy of Sciences, Kunming, 650223 China; 10 KIZ/CUHK Joint Laboratory of Bioresources and Molecular Research in Common Diseases, Kunming, 650223 China

**Keywords:** Long noncoding RNA, Hypoxia, Carbonic anhydrase 9, Cancer, Hypoxia-induced factor, DNA methylation, Alternative promoter, Alternative splicing

## Abstract

**Background:**

Long noncoding RNAs (lncRNAs) are important regulators in tumor progression. However, their biological functions and underlying mechanisms in hypoxia adaptation remain largely unclear.

**Results:**

Here, we established a correlation between a Chr3q29-derived lncRNA gene and tongue squamous carcinoma (TSCC) by genome-wide analyses. Using RACE, we determined that two novel variants of this lncRNA gene are generated in TSCC, namely *LINC00887_TSCC_short* (*887S*) and *LINC00887_TSCC_long* (*887L*). RNA-sequencing in *887S* or *887L* loss-of-function cells identified their common downstream target as *Carbonic Anhydrase IX* (*CA9*), a gene known to be upregulated by hypoxia during tumor progression. Mechanistically, our results showed that the hypoxia-augmented *887S* and constitutively expressed *887L* functioned in opposite directions on tumor progression through the common target *CA9*. Upon normoxia, *887S* and *887L* interacted. Upon hypoxia, the two variants were separated. Each RNA recognized and bound to their responsive DNA cis-acting elements on *CA9* promoter: *887L* activated *CA9*’s transcription through recruiting HIF1α, while *887S* suppressed *CA9* through DNMT1-mediated DNA methylation.

**Conclusions:**

We provided hypoxia-permitted functions of two antagonistic lncRNA variants to fine control the hypoxia adaptation through CA9.

**Supplementary Information:**

The online version contains supplementary material available at 10.1186/s12915-021-01112-2.

## Background

Oxygen is of fundamental importance for human cells. Reduced level of oxygen or hypoxia, generated by external and internal changes, may cause pathological consequences regionally and globally, or even life-threatening conditions. Tumor hypoxia, resulted from the rapid growth of solid tumor cells, is one of the most commonly observed features of tumor microenvironment [1–6]. To survive and gain growth advantage over normal cells, tumor cells developed a series of adaptive mechanisms under hypoxia. Hypoxia-induced factor alpha (HIFα) plays a key role in tumor hypoxia adaptation [2, 4, 6]. When the oxygen level falls, accumulated HIFα proteins will translocate to the nucleus, bind to the HIF response element (HRE) located in the 5’ regulatory regions of their downstream target genes and activate a broad range of gene transcription events [2, 4]. The HIFα downstream genes include *carbonic anhydrase* 9 (*CA9*), and many other oncoproteins [2, 4, 7–10]. Despite the positive influences on tumor progression, it has been proposed that prolonged activation of HIF may be potentially deleterious to the tumor cells per se and chronic reduction of HIFα proteins is to avoid “maladaptive” or “potentially deleterious effects” resulted from over-activation of the HIF pathway [11, 12]. Therefore, the fine control of HIFα downstream genes is critical for tumor cell progression.

Long noncoding RNAs (lncRNAs) represent a group of regulatory RNAs that are larger than 200 nucleotides. According to the GENCODE database (GRch38, version 32), more than 57,935 human lncRNAs have been identified and this number is still climbing due to the development of advanced sequencing techniques [13, 14]. Increasing evidence has shown that lncRNAs are important regulators in almost all the physical and pathological events, including tumor progression [15–18]. However, relative limited investigations have revealed the functions and molecular mechanisms of lncRNAs in tumor hypoxia [19, 20]. It remains to be a challenge to unveil the comprehensive picture of lncRNAs’ engagement in hypoxia adaptation.

In the present study, we identified that the activity of CA9, a well-known HIF1α target gene and oncoprotein, is regulated through a pair of functionally antagonistic lncRNA variants in tongue squamous cell carcinoma (TSCC). Our results shed light on an lncRNA-directed mechanism to fine controlled oncoprotein CA9’s expression during hypoxia adaptation.

## Results

### Identification of *LINC00887* as a hypoxia-associated lncRNA in TSCC

**Fig. 1:**
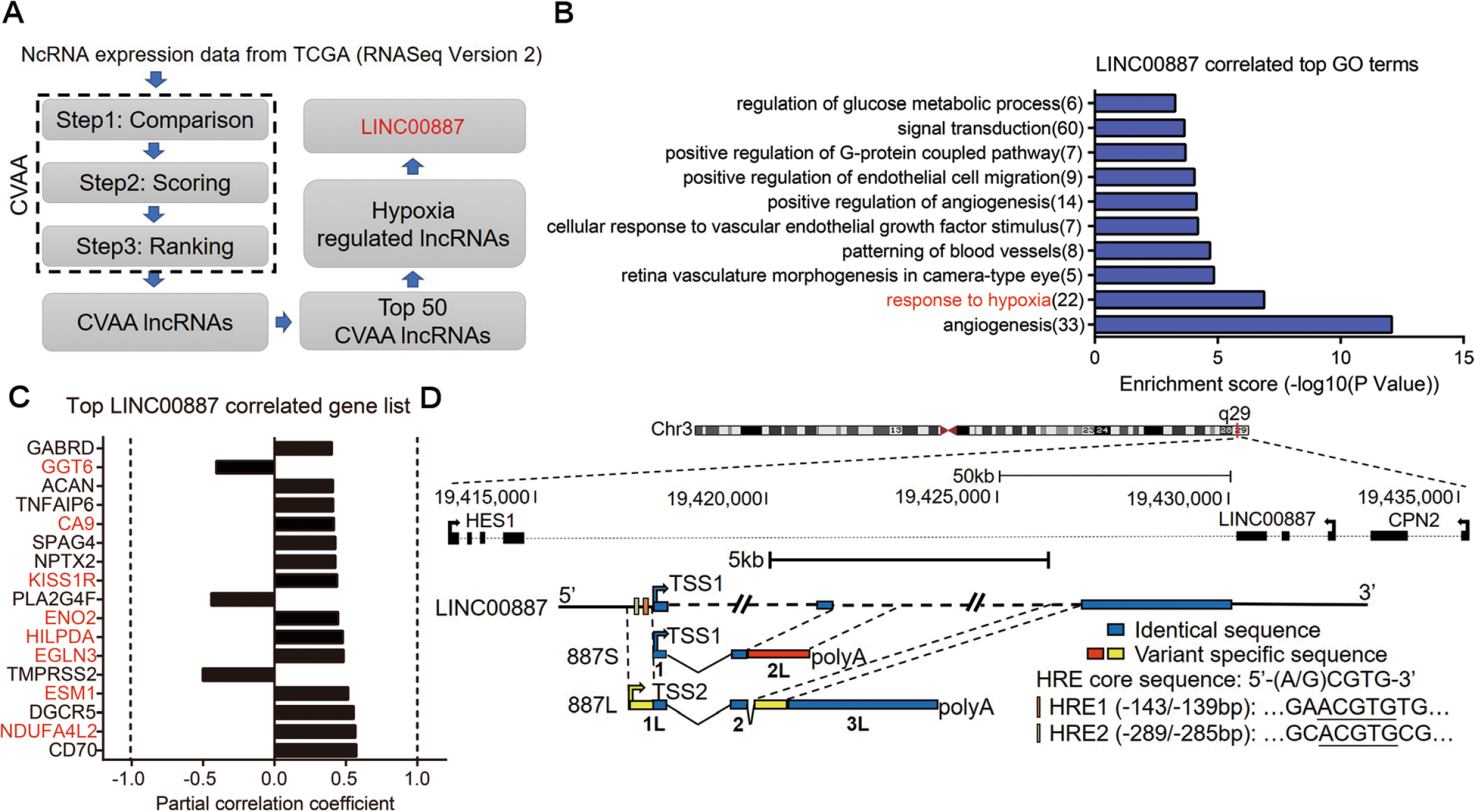
Identification of a hypoxia-regulated CVAA lncRNA gene, *LINC00887*, which generated two lncRNA variants *887S* and *887L* in TSCC. **A** A schematic view of experimental outline for screening hypoxia-regulated CVAA lncRNAs. **B** Top 10 biological processes that are related to *LINC00887* and significantly enriched by Gene Ontology (GO) analysis. **C** *LINC00887-*correlated genes according to Partially Correlation Analysis (PCA) analysis (*P*<0.05). Hypoxia-associated genes are marked in red. **D** A schematic comparation of *887S*, *887L*, and *LINC00887*

In order to identify the tumor-associated lncRNAs that might play roles in hypoxia adaptation, we first identified lncRNAs that were dysregulated in cancer patients by cross-value association analysis (CVAA), a normalization-free and nonparametric method that we recently developed [21], and searched those resulted lncRNAs whose expression levels were regulated by hypoxia (Fig. 1A). Briefly, we applied CVAA to the RNA-seq data-sets of 5540 patient samples retrieved from The Cancer Genome Atlas (TCGA), including 4907 tumor specimens from 13 types of primary solid tumor and 631 normal specimens from matching tissue type. The identified lncRNAs that were dysregulated in pan-cancer will be further referred as CVAA lncRNAs in this work. Next, to identify the hypoxia-regulated lncRNAs from the resulted top 50 CVAA lncRNAs, we searched the published reports on PubMed and found 6 CVAA lncRNAs whose expression levels were regulated by hypoxia treatment (Supplementary table 1, [22–31]). Among which, the involvement of *LINC00887* in tumor hypoxia is currently unknown despite that it was initially reported to be induced by hypoxia in renal epithelial cell [24]. We then focused on *LINC00887* and performed Partial Correlation Analysis (PCA) and Gene Ontology (GO) analysis using RNA-seq data-sets retrieved from TCGA. PCA and GO analysis showed that the function of *LINC00887* was highly enriched in “response to hypoxia” (Fig. 1B). In addition, PCA analysis established significant correlation between *LINC00887* and 17 genes (Fig. 1C). All of these genes have been shown to play important roles in tumor progression [32–47]. Interestingly, 8 of these 17 genes have been identified as hypoxia-associated genes, including *CA9*, *gamma-glutamyltransferase 6* (*GGT6*), *KISS1 receptor* (*KISS1R*), *enolase 2* (*ENO2*), *hypoxia-inducible lipid droplet-associated protein* (*HILPDA*), *EGL-9 family hypoxia inducible factor 3* (*EGLN3*), *endothelial cell specific molecule 1* (*ESM1*), *and NDUFA4 mitochondrial complex-associated like 2* (*NDUFA4L2*) (Fig. 1C). Besides GGT6, that is negatively correlated with *LINC00887*, the other 7 hypoxia-associated genes showed to be positively correlated with *LINC00887*.

We also performed a series of examinations and built a strong link between *LINC00887* and TSCC: *LINC00887* is derived from chromosome 3q29, a genomic region that is highly associated with squamous cancer by integrative large-scale analysis [48, 49]. Consistently, by using a set of cultured tumor cells for the expression levels of the top 10 CVAA lncRNAs, we found that *LINC00887* was the only lncRNA to be upregulated in TSCC cells (including TSCC9, TSCC15, and TSCC25) (Fig. S1A). In addition, by analyzing the RNA-seq data from TCGA retrieved TSCC patients, we found that the expression level of *LINC00887* was dramatically increased in TSCC and the upregulation of *LINC00887* was positively correlated with poor patient survival rate (Fig. S1B, C). All together, our data suggested that *LINC00887* might be a critical regulator to hypoxia response in TSCC.

### *LINC00887* generates two variants, *887S* and *887L*, in TSCC

Although *LINC00887*, also named as *linc-ATP13A4-8* and *HEIRCC* [24, 50], has been reported as a long intergenic noncoding RNA derived from the antisense strand of chromosome (Chr.) 3q29 (Fig. 1D, upper panel), the genomic structure of this lncRNA has not yet been characterized. By performing 5′ and 3′ rapid-amplification of cDNA ends (RACE) assays, we found two previously unidentified variants of *LINC00887* in TSCC (Fig. S2A-D). Due to their different length and the initial identification from TSCC, we designated these two variants as *LINC00887_TSCC_Short* (*887S*, 1593nt) and *LINC00887_TSCC_Long* (*887L*, 4202nt). For simplicity, we used “*887S*” and “*887L*” in the following manuscript and all datasets. The genome structure difference between *887S* and *887L* suggests that these two variants are generated by combined effects of alternative promoter (AP) selection and alternative splicing (AS). Compared with *LINC00887* shown in NCBI database (Fig. 1D and S2E), *887S* has an extra 1118bp at the 3′ of exon2 (named as exon2L for *887S*) (Fig. 1D and S2E), and *887L* has an extra 405bp at the 5′ of exon1 (named as exon1L for *887L*) and an extra 776bp at the 5′ of exon3 (named as exon3L for *887L*) (Fig. 1D and S2E).

Next, we conducted RT-PCR assay by using a set of primers that specifically recognized *887S*, *887L*, or *LINC00887* based on NCBI database (Fig. S2C, D). The resulted PCR products were gel-purified and analyzed by Sanger sequencing. These results showed that the existence of *887S* and *887L*, but not the previously annotated *LINC00887* in NCBI, in all three tested TSCC cell lines (TSCC9, TSCC15, and TSCC25; Fig. S2D). Using specific probes for *887S* and *887L*, our Northern blot results showed that the two investigated RNAs were relatively abundant in both TSCC15 and TSCC25 cell lines. In contrast, both RNAs were either undetectable or showed much weak signal in 293T cells (Fig. S2F-H). *LINC00887* has additional 17 and 10 variants shown in LNCipedia and Ensemble databases, respectively. To test the existence of these variants, we designed specific primers for their common exons. In contrast to *887S* and *887L* in the same experiment, our RT-qPCR results showed that none of these variants, except *LINC00887* variants 15 and 16, was expressed in TSCC (Fig. S2F, I). Although variants 15 and 16 were expressed in TSCC, the net expression levels of both variants were only about 1/3 of the expression level of *887S* or *887L* (Fig. S2F, I). These results indicated that *887S* and *887L* were two novel and predominate variants in TSCC cells. We further confirmed the existence of *887S* and *887L* in TSCC patient samples (Fig. S3C). Protein-coding potential analysis by CPC (coding potential calculator, http://cpc.cbi.pku.edu.cn) showed that both *887S* and *887L* had no typical protein-coding ORF (Fig. S4A). All together, our results suggested that the previously reported lncRNA *LINC00887* can generate two lncRNA variants, *887S* and *887L*, in TSCC.

### LncRNA-*887S* and LncRNA*-887L* exhibit differential response to hypoxia

**Fig. 2:**
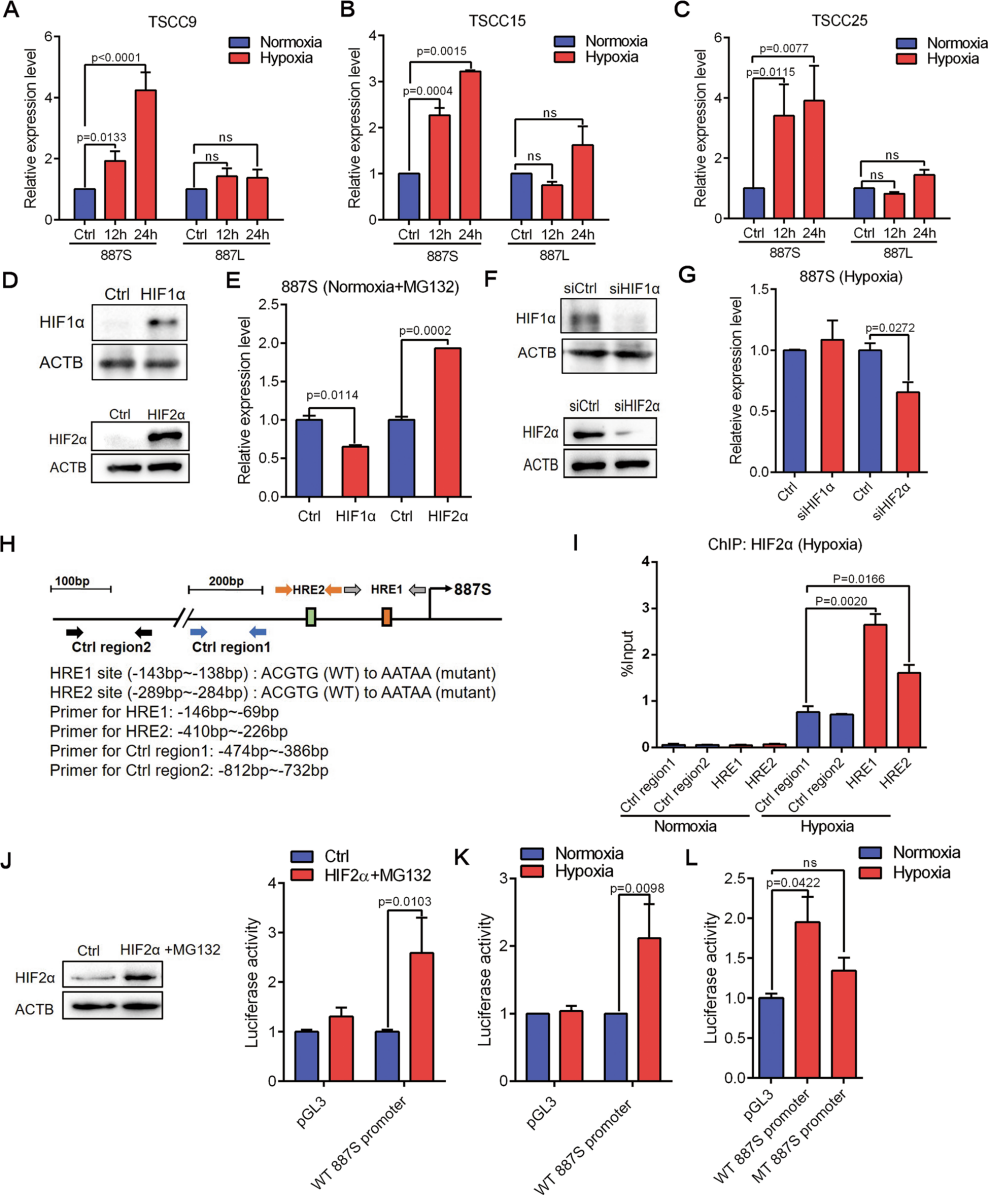
*887S* and *887L* are differently modulated upon hypoxia. **A**–**C** Relative expression levels of *887S* and *887L* in TSCC9 (**A**), TSCC15 (**B**), and TSCC25 (**C**) in the presence of normoxia or hypoxia (*n*=3). **D** Overexpression efficiency of HIF1α (upper panel) or HIF2α (lower panel) with treatment of MG132 (10μM, 8h) in TSCC15 cells. (*n*=3). **E** Relative expression levels of *887S* in response to HIF1α or HIF2α overexpression in the presence of MG132 (*n*=3). **F** Knockdown efficiency of HIF1α siRNA (upper panel) or HIF2α siRNA (lower panel) under hypoxia (*n*=3). **G** Relative expression levels of *887S* in the presence of HIF1α or HIF2α siRNAs under hypoxia (*n*=3). **H** A schematic view of the 5’ regulatory regions of *887S* with the relative location of HREs, wide-type, and mutant sequences of HREs, and the primers used in ChIP assays. **I** ChIP assay on the indicated regions of *887S* promoter upon normoxia and hypoxia (*n*=3). **J** Overexpression efficiency of HIF2α with treatment of MG132 (10μM, 8h) in TSCC15 cells (left panel) and activity change of the pGL3 control (pGL3) or wild type (WT) *887S* promoter after modulation of HIF2α (right panel). (*n*=4). **K** Activity change of the pGL3 control (pGL3) or wild type (WT) *887S* promoter after modulation of oxygen levels (*n*=4). **L** Activity change of the WT or mutant (MT) *887S* promoter in the presence of normoxia and hypoxia (*n*=4). Data are shown as means ± SEMs. *P* values are calculated using Student’s *t* test

The differential transcriptional start sites (TSSs) of *887S* and *887L* resulted in an inclusion of two putative HRE sites exclusively located in the promoter of *887S* (Fig. 1D, lower panel). In contrast, the promoter of *887L* which has an extra 5′ sequences at the first exon did not contain the HRE sites (Fig. 1D, lower panel). HRE (5′-A/GCGTG-3′) is the cis-acting DNA element receiving the regulation of HIF family members [51]. Consistently, *887S*, instead of *887L*, could be significantly induced when the TSCC9, TSCC15, and TSCC25 cells were cultured under 1% O_2_ (Fig. 2A–C, S3A, B). HIF1α and HIF2α are accumulated under hypoxia or nomoxia but in the presence of MG132 to inhibit the proteasome degradation pathway [51, 52]. Our further experiments showed that the expression level of *887S* was positively regulated by HIF2α, instead of HIF1α (Fig. 2D–G). Moreover, upon hypoxia, HIF2α was recruited to both HRE sites of *887S* by ChIP assay (Fig. 2H, I). The luciferase reporter assay showed that either hypoxia or overexpression of HIF2α (in the presence of MG132) could enhance the activity of the wild type *887S* promoter (Fig. 2J, K), which was abolished when we mutated the two HRE sites from 5′-CGTG-3′ to 5′-ATAA-3′ (Fig. 2L). These data suggested that the expression level of lncRNA*-887L* is hypoxia-independent, and lncRNA*-887S* is a HIF2α-inducible transcript upon hypoxia.

### LncRNA-*887S* and LncRNA*-887L* play antagonistic roles in TSCC through regulating CA9 in opposite directions

In order to investigate the subcellular location of lncRNA-*887S* and *887L*, we performed RNA fluorescence in situ hybridization (FISH) and fractionationing assay in TSCC15 cells. Our results revealed a predominant nuclear expression pattern of both transcripts under both normoxia and hypoxia (Fig. S4B-D).

To further investigate the two lncRNAs’ biological functions, we took advantage of the identification that *887S* was induced by hypoxia via HRE sites and established CRISPR-Cas9-mediated HRE mutant TSCC15 lines (HREmut1 and HREmut2) that could effectively abolish the induction of *887S* upon hypoxia, without affecting the expression level of *887S* under normoxia (Fig. S5A). Although the HRE mutation is also located within the first exon of *887L*, the expression levels of *887L* were not altered detected by multiple primer sets targeting the unique *887L* exons (Fig. S5B, C). Therefore, the HRE mutant lines allowed us to investigate the role of *887S* transcript exclusively for the hypoxic condition. For *887L*, we designed two independent small hairpin RNAs (shRNAs) targeting the exon3L of *887L* (sh*887L1* and sh*887L2*) (Fig. S5D).

**Fig. 3:**
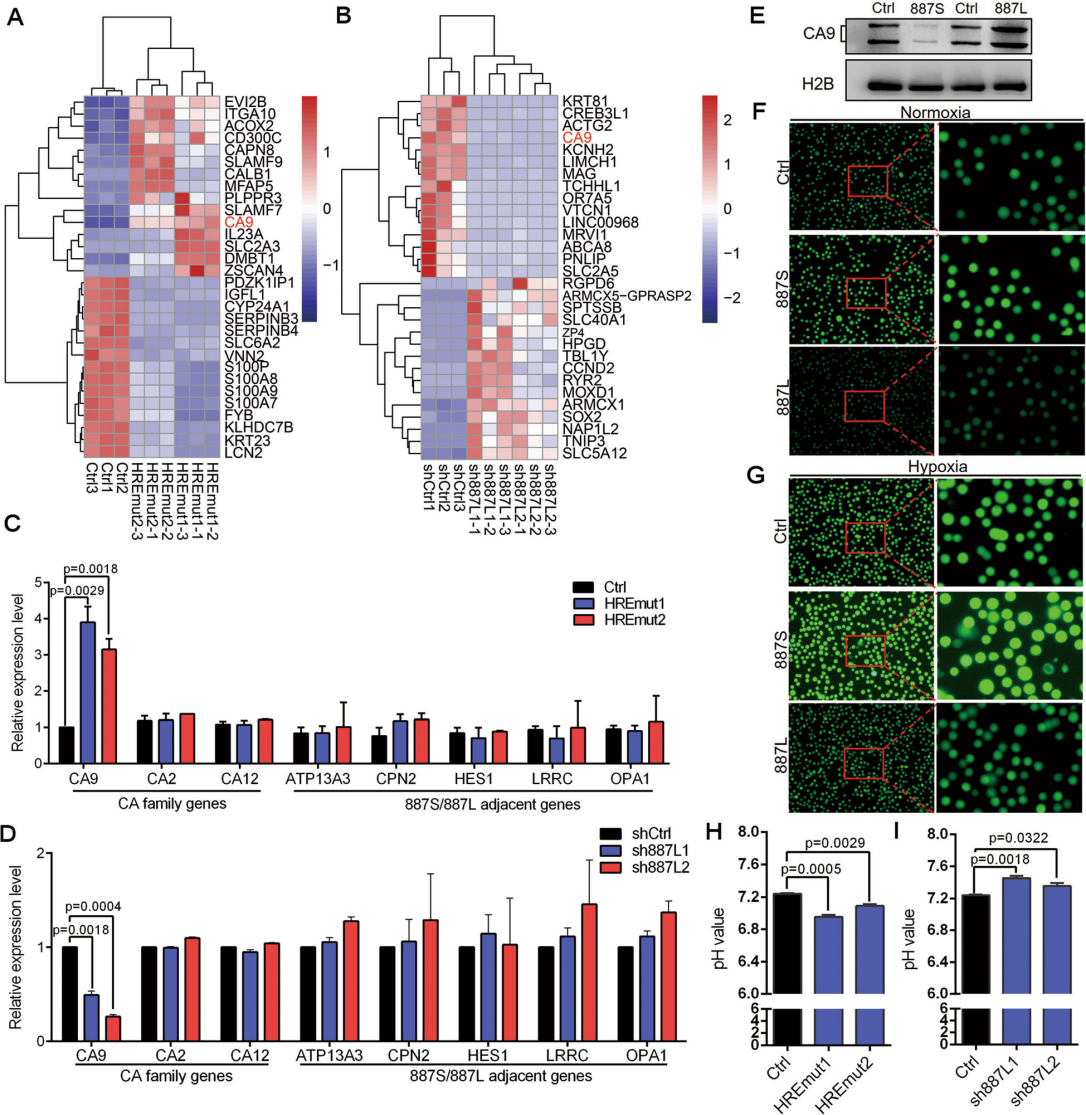
*887S* and *887L* antagonistically regulate the expression and activity of CA9. **A** The top 15 upregulated or downregulated genes identified by RNA-seq in the two indicated *887S* knockout cell lines (HREmut1 and HREmut2) and the corresponding control lines under hypoxic condition. **B** The top 15 upregulated or downregulated genes identified by RNA-seq in the two indicated *887L* knockdown cell lines (sh*887L*1 and sh*887L*2) and the corresponding control lines under normoxic condition. **C**, **D** Relative expression levels of the indicated CA family genes and *887S*/*887L* adjacent genes (*ATP13A3*, *CPN2*, *HES1*, *LRRC*, *OPA1*) in *887S* knockout cells under hypoxic condition (**C**) or *887L* knockdown cells under normoxic condition (**D**) (*n*=3). **E** The protein levels of CA9 in *887S* or *887L* overexpression cells (*n*=3). Related to Figure S5E, F. **F**, **G** Images of the fluorescence intensity detected by BCECF-AM assay in the indicated *887S* or *887L* overexpression cells in the presence of normoxia (**F**) and hypoxia (**G**). **H** Extracellular pH values of the indicated control (Ctrl) and *887S* knockout cells under hypoxic condition. **I** Extracellular pH values of the indicated control (Ctrl) and *887L* knockdown cells under hypoxic condition. Data are shown as means ± SEMs. *P* values are calculated using Student’s *t* test

The nuclear expression suggested that *887S* and *887L* may function in transcriptional regulation. In order to identify the *887S* and *887L* regulated downstream genes, we performed RNA-sequencing (RNA-seq) in the *887S* knockout cells (HREmut1 and HREmut2), *887L* knockdown cells (sh*887L*1 and sh*887L*2), and their corresponding control cells. When compared with the corresponding controls, 318 or 855 genes were identified as common dysregulated genes for HREmut1 and HREmut2, or sh*887L*1 and sh*887L*2, respectively. Interestingly, among the top regulated genes, *CA9*, one of the 8 hypoxia-associated genes we identified by PCA (Fig. 1C), was shown to be a common downstream target of *887S* and *887L* in an opposite regulatory manner (Fig. 3A, B).

*CA9*, a well-known hypoxia-induced gene, plays critical roles in promoting tumor progression and hypoxia adaptation [7]. Our analysis showed that the upregulation of CA9 was correlated with the poor survival rate in TCGA retrieved TSCC patients (Fig. S6A, B). The effect of CA9 on TSCC cells was also tested by colony formation and transwell assays. As shown in Fig. S6C-G, we found that knockdown of CA9 resulted in a remarkable decrease of tumor cell progression in TSCC15 cells. These results indicated that CA9 acted as a tumor promoter in TSCC. After validating the in-house RNA-seq data by RT-qPCR assay (Fig. 3C, D, and S7A), we performed western blot assay and showed that the protein level of CA9 was also reversely regulated by *887S* and *887L* (Fig. 3E, and S5E, F). CA9 is a zinc metalloenzyme that facilitates tumor acidification through hydration of carbon dioxide [8, 53]. To answer the question whether the function of CA9 as a pH modulator was altered as a result of the dysregulated mRNA and protein levels in either *887S* or *887L* modulated cells, we performed the BCECF-AM assay. As expected, our results showed that *887S* decreased the value of intracellular pH, while *887L* increased the value of intracellular pH (Figs. 3F, G, and S8). In consent with the BCECF-AM assay, the extracellular pH value was decreased when *887S* was knocked out but increased when *887L* was knocked down (Fig. 3H, I).

CA9 belongs to carbonic anhydrase family, which is composed of 16 members in human. Distinct from CA9, the other CA members are predominately expressed in normal tissues [54, 55]. Although TSCC tumor samples and TSCC15 cells also express CA2 and CA12, our in-house RNA-seq data showed that the expression levels of CA2 and CA12 were not altered when the expression of **887S** or **887L** was modulated. These results were further validated by the RT-qPCR tests (Fig. 3C, D). In addition, the direct (*HES1*, *CPN2*, as shown in Fig. 1A) and far neighboring genes of *887S*/*887L* on Chr.3 (**ATP13A,** **LRRC,** and **OPA1**) were not regulated by the two lncRNAs (Fig. 3C, D). Together, our results indicated that **887S** and **887L** exhibited a strong specific regulation on CA9 among carbonic anhydrase family in TSCC.

**Fig. 4:**
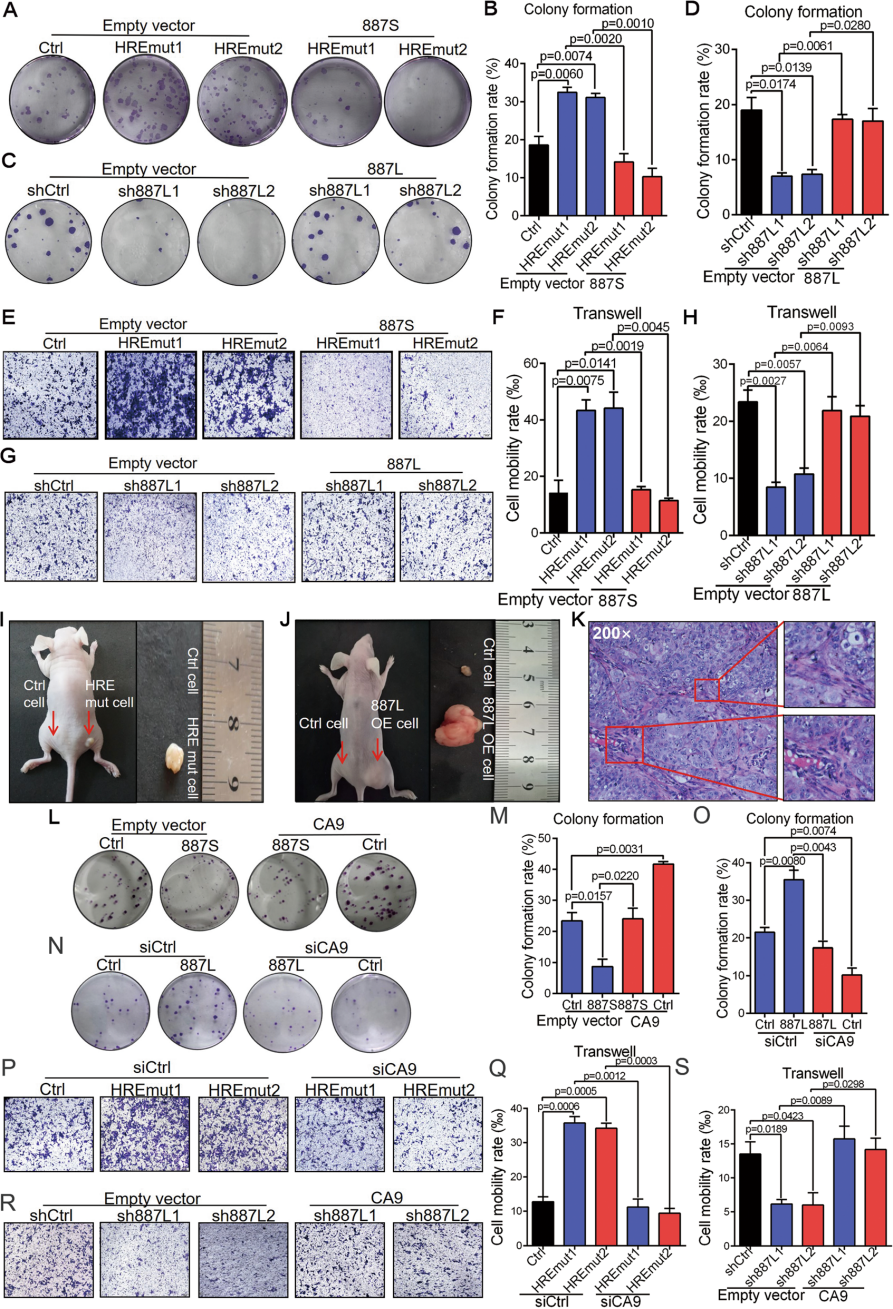
*887S* and *887L* drive TSCC progression in opposite directions through CA9 under hypoxia. **A** Representative images of colony formation assay in *887S* knockout cells in the presence of control or *887S* overexpressed plasmid. **B** Statistical analysis according to **A** (*n*=3). Related to Figure S5G. **C** Representative images of colony formation assay in *887L* knockdown cells in the presence of control or *887L* overexpressed plasmid. **D** Statistical analysis according to **C** (*n*=3). Related to Figure S5H. **E** Representative images of transwell assay in *887S* knockout cells in the presence of control or *887S* overexpressed plasmid. **F** Statistical analysis according to **E** (*n*=3). Related to Figure S5G. **G** Representative images of transwell assay in *887L* knockdown cells in the presence of control or *887L* overexpressed plasmid. **H** Statistical analysis according to **G** (*n*=3). Related to Figure S5H. **I**, **J** Representative images of xenografts from Balb/c (nu/nu) mice injected with *887S* knockout (**I**) or *887L* overexpressed (**J**) TSCC15 cells. The arrows denote tumors in situ. **K** HE staining image of tumor specimen dissected from the xenograft mice. The enlarged regions indicate the keratinized feature of TSCC15. **L** Representative images of colony formation assay in *887S* overexpressed cells in the presence of control or CA9-overexpressed plasmid. **M** Statistical analysis according to **L** (*n*=3). Related to Figure S7B. **N** Representative images of colony formation assay in *887L* overexpressed cells in the presence of control or siCA9. **O** Statistical analysis according to **N** (*n*=3). Related to Figure S7C. **P** Representative images of transwell assay in *887S* knockout cells in the presence of control or siCA9. **Q** Statistical analysis according to **P** (*n*=3). **R** Representative images of transwell assay in *887L* knockdown cells in the presence of control or CA9-overexpressed plasmid. **S** Statistical analysis according to **R** (*n*=3). Data are shown as means ± SEMs. *P* values are calculated using Student’s *t* test

Next, we wanted to explore the biological roles of *887S* and *887L* on tumor progression under hypoxia. Knockout of *887S* remarkably promoted tumor progression in TSCC15 cells observed by colony formation and transwell assays (Fig. 4A, B, E, F, and S5G). On the contrary, knockdown of *887L* significantly inhibited tumor progression in TSCC15 cells (Fig. 4C, D, G, H, and S5H). To test whether the above identified roles were dependent on the two investigated lncRNAs, we overexpressed the *887S*- or *887L*-expressing plasmid in their corresponding knockout or knockdown cells and found that both overexpressions could significantly reverse the effects (Fig. 4A–H, and S5G, H). Consistently, modulating the expression levels of *887S* and *887L* dramatically affected TSCC growth in the opposite direction by xenograft experiments (Fig. 4I–K and S9).

Given that *CA9* is a common downstream target of *887S* and *887L*, we sought to evaluate whether *887S* and *887L* regulated tumor cell progression via CA9. We introduced CA9-expression plasmid in *887S* stable overexpression cells and found that the overexpression of CA9 completely blocked the effect caused by *887S* (Fig. 4L, M, and S7B). Vice versa, knockdown of CA9 in *887S* stable knockout cells also completely reversed the effects caused by *887S* (Fig. 4P, Q). In addition, similar rescue effect was observed when we modulated the expression level of CA9 in *887L* stably overexpression cells (Fig. 4N, O, and S7C) or *887L* knockdown cells (Fig. 4R, S). To further validate the observed effects in *887S* knockout cell lines, we also conducted ASO-mediated *887S* knockdown assays (Fig. S10A). Our results showed that *887S* ASO enhanced the promoter activity of CA9, increased the expression levels of CA9 mRNA and protein, and promoted TSCC15 cell proliferation and migration ability (Fig. S7E, F, and S10B-H). Together, our data suggested that, although *887S* and *887L* are both upregulated in TSCC, these two transcripts play antagonistic roles in TSCC through CA9.

### LncRNA-*887L* is required for the HIF1α-induced activation of *CA9* in TSCC

**Fig. 5:**
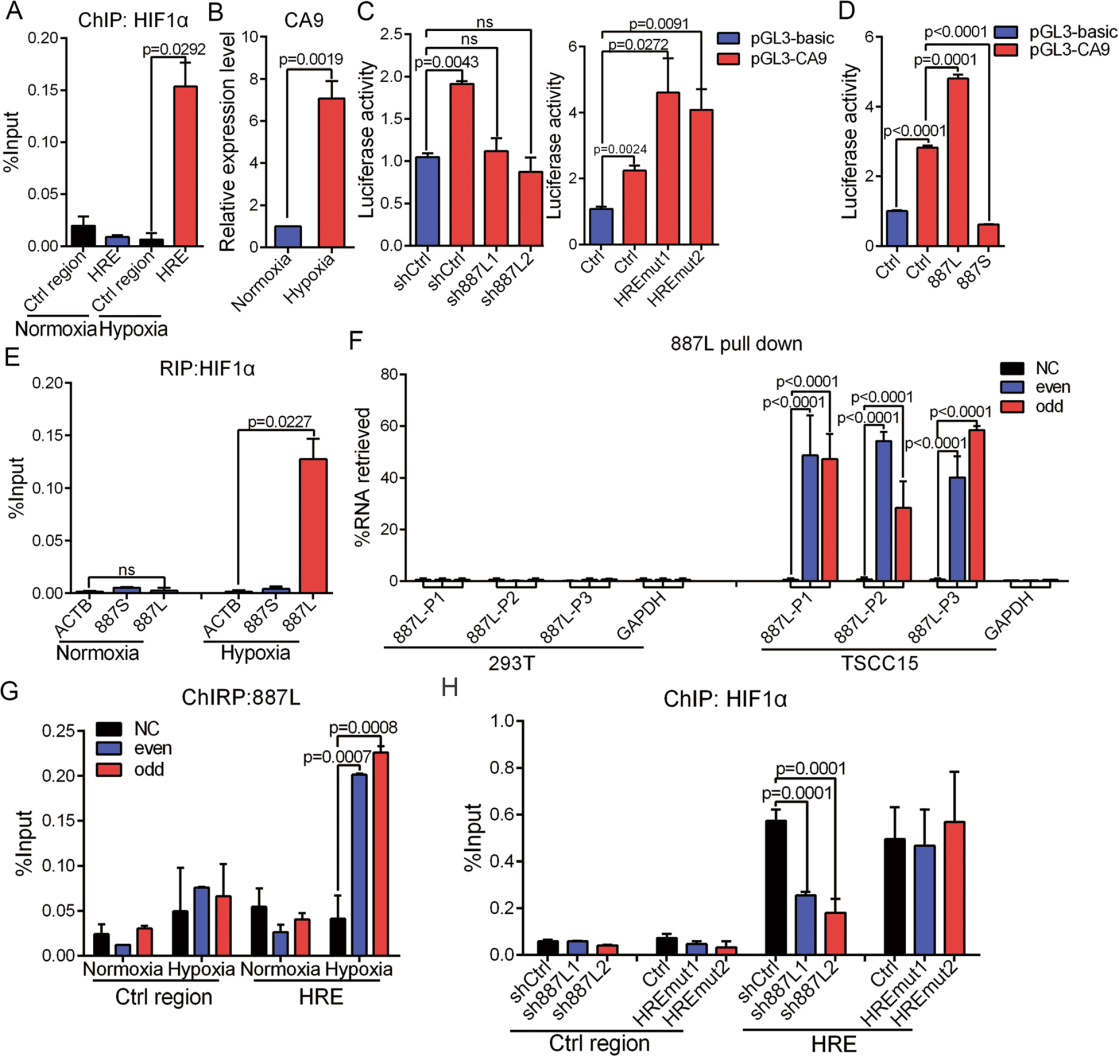
*887L* is required for the HIF1α-induced activation of CA9 under hypoxia. **A** HIF1α ChIP assay on the indicated HRE site and control region on *CA9* promoter upon normoxia and hypoxia (*n*=3). **B** Relative expression levels of CA9 under normoxia and hypoxia (*n*=3). **C** Activity changes of *CA9* promoter in the *887L* knockdown cells or *887S* knockout cells (*n*=3). **D** Activity change of *CA9* promoter in the *887L* overexpressed cells or *887S* overexpressed cells (*n*=3). **E** HIF1α RIP assay showed the immunoprecipitation of the indicated β-actin (ACTB), *887S*, and *887L* RNAs upon normoxia and hypoxia (*n*=3). **F** Capture of *887L* RNA by RNA pull-down assay in 293T cells, that do not express *887L*, and TSCC15 cells. NC: non-probe control; even and odd: two separated pools of *887L* probes containing either even or odd numbered probes based on their positions along the *887L* sequence. *887L*-P1, *887L*-P2, and *887L*-P3: primers for *887L* detection; GAPDH: primers for GAPDH detection. (*n*=2). **G** ChIRP assay showed the association of the *887L* RNA with the *CA9*’s HRE site under normoxia and hypoxia (*n*=2). **H** Recruitment of HIF1α to the control region and HRE site of *CA9* promoter in *887S* knockout and *887L* knockdown cells (*n*=3). Data are shown as means ± SEMs. *P* values are calculated using Student’s *t* test

Previous work has reported that *CA9* is transcriptionally activated by HIF1α in a broad spectrum of tumor cells [56–58]. In TSCC15 cells, we found that CA9 was also upregulated by HIF1α upon hypoxia when HIF1α was recruited to the HRE site of *CA9* promoter (Fig. 5A, B). Furthermore, we constructed a luciferase reporter driven by *CA9* promoter and performed luciferase assay. Our data showed that *887S* and *887L* could either inhibit or enhance the hypoxia-induced *CA9* promoter activity, respectively (Fig. 5C, D), suggesting that both *887S* and *887L* regulated *CA9* at the transcriptional level. Interestingly, our RNA immunoprecipitation (RIP) assay showed that only *887L* interacted with HIF1α (Fig. 5E).

To test whether the *887L* could be recruited to the HRE site of *CA9*, we synthesized antisense probes specifically targeting *887L* and performed chromatin isolation by RNA pull-down (ChIRP) assays. The RT-qPCR following RNA pull-down was first performed to prove the specificity and efficiency of the *887L* probes. Both the odd and even sets of *887L* probes were shown to be highly effective in TSCC15 (Fig. 5F). In contrast, no PCR product was amplified using the control probes in TSCC15 cells or using *887L* probes in the 293T cells that did not express *887L* (Fig. 5F). These results indicated that the *887L* probes were highly specific and effective. Then, we examined the *887L*-associated chromatin and found that hypoxia induced a specific association of *887L* RNA with the HRE site of *CA9* (Fig. 5G). Moreover, the hypoxia-induced recruitment of HIF1α was found to be dramatically decreased when *887L* was knocked down (Fig. 5H). In contrast, *887S* knockouts showed no impact on the HIF1α recruitment (Fig. 5H). All together, our results indicated that *887L* is required for the HIF1α-induced activation of CA9 in TSCC15 cells.

### LncRNA-*887S* negatively regulates *CA9* through DNA methyltransferase 1 (DNMT1)-mediated DNA methylation

**Fig. 6:**
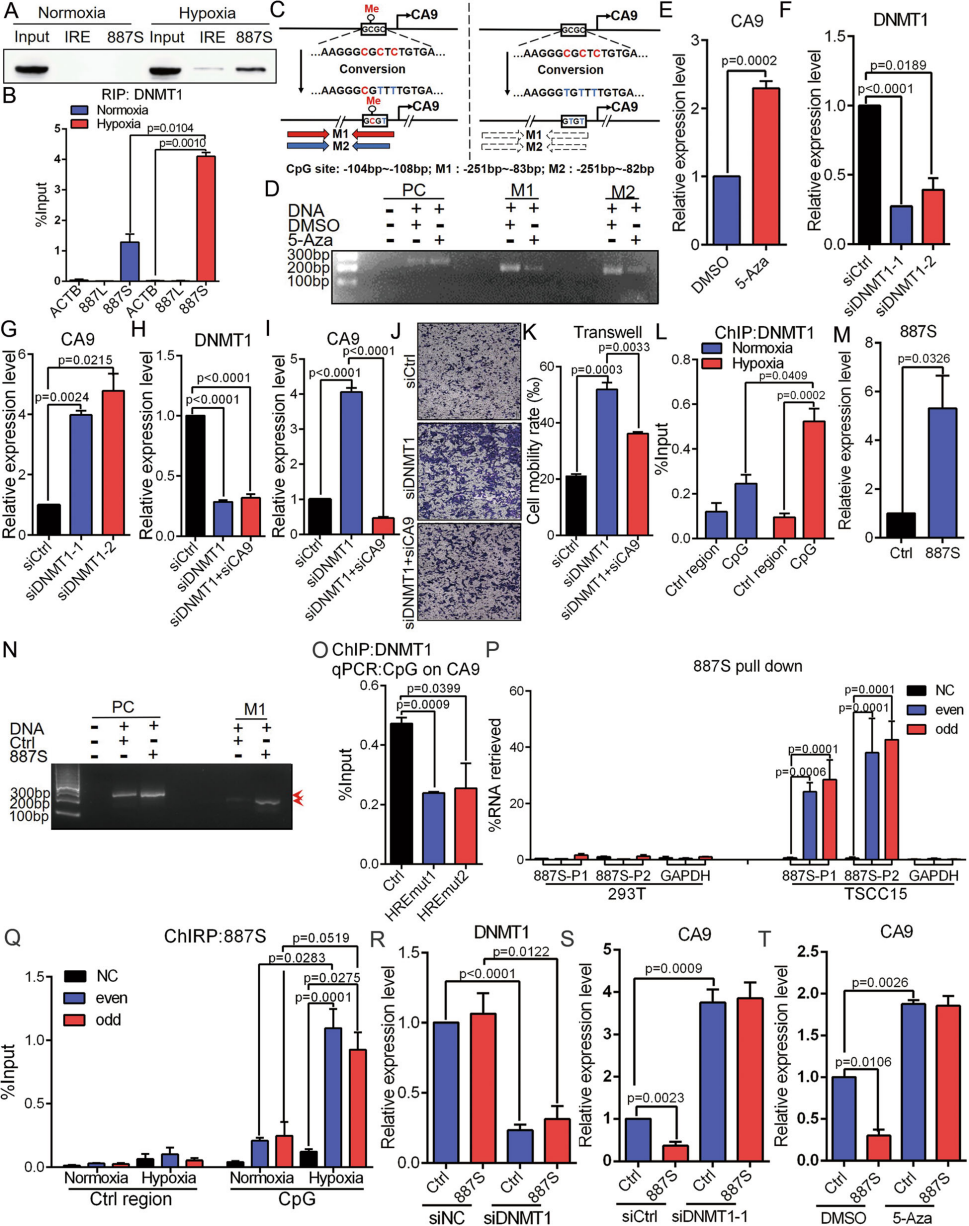
*887S* inhibits CA9 expression through regulating DNMT1-mediated DNA methylation upon hypoxia. **A** DNMT1 was identified as *887S*-associated protein in a hypoxia-dependent manner, by RNA pull down and Western blot. IRE: a control RNA provided by Pierce RNA 3′ End Desthiobiotinylation kit. Related to Figure S11. **B** DNMT1 RIP assay showed *887S*, instead of *887L* RNA was immunoprecipitated under hypoxia (*n*=3). **C** A schematic view of the process to detect methylation status of the CpG dinucleotides. M1 and M2: two primer sets to amplify the converted DNA sequence of CpG site on CA9 promoter. **D** Representative image of methylation status of the CpG site on CA9 promoter with or without 5-Aza treatment by the indicated PCR primer sets. PC: a primer set to detect the C to T conversion which is provided by the MSP kit. **E** Relative expression levels of CA9 in TSCC15 cells in the presence of carrier (DMSO) or 5-Aza (*n*=3). **F**, **G** Effects of the indicated DNMT1 siRNAs on DNMT1 (**F**) and CA9 (**G**) mRNA level (*n*=3). **H**, **I** Relative expression levels of DNMT1 (**H**) or CA9 (**I**) under the indicated treatments (*n*=3). **J**, **K** Transwell assay showed that knockdown of CA9 significantly inhibited the effect of DNMT1 knockdown in TSCC15 cells (*n*=3). **L** DNMT1 ChIP assay on the indicated CpG site and control region of *CA9* upon normoxia and hypoxia (*n*=3). **M** Overexpression efficiency of *887S* (*n*=3). **N** Representative image of methylation status of the CpG site on CA9 promoter detected by M1 in the control or *887S*-overexpressed cells. **O** DNMT1 ChIP assay on the CpG site of CA9 promoter in *887S* knockout cells under hypoxia (*n*=3). **P** Capture of *887S* RNA by RNA pull-down assays in 293T cells, that do not express *887S*, and TSCC15 cells. NC: non-probe control; even and odd: two separated pools of *887S* probes containing either even or odd numbered probes based on their positions along the *887S* sequence. *887S*-P1 and *887S*-P2: primers for *887S* detection; GAPDH, primers for GAPDH detection. (*n*=2). **Q** ChIRP assay showed the association of the *887S* RNA with the *CA9*’s CpG site under normoxia and hypoxia (*n*=2). **R**, **S** Relative expression level of DNMT1 (**R**) or CA9 (**S**) in control and *887S* overexpressed TSCC15 in the presence of siDNMT1 (*n*=3). **T** Relative expression levels of CA9 in Ctrl and *887S* overexpressed TSCC15 cells in the presence of carrier (DMSO) or 5-Aza (*n*=3). Data are shown as means ± SEMs. *P* values are calculated using Student’s *t* test

In Fig. 5E, H, we have showed that *887S* negatively regulated CA9 in a HIF1α-independent manner. To further explore the molecular mechanism of *887S*, we performed an RNA pull-down assay followed by mass spectrometry to identify the *887S*-associated proteins. Among all the candidates, we focus on DNMT1 because a DNA methylation-involved regulation on CA9 has been recently suggested in a subset of tumor cells [59]. The interaction of *887S*:DNMT1 was further confirmed by RNA immunoprecipitation (RIP) assay and RNA pull-down followed by western blot assay (Fig. 6A, B and S11). DNA methylation site was mapped to a CpG dinucleotide-containing region located at upstream of the TSS of *CA9* with unknown mechanisms [59]. It has been known that DNMT1 regulates DNA methylation presumably in mammalian somatic cells, and its activity is sufficient to methylate CpG-poor regions [60–62]. These features make DNMT1 as a promising candidate of *CA9* methylation.

In order to investigate whether DNA methylation affects CA9’s expression in TSCC, we performed methylation specific PCR (MSP) assay and found a detectable DNA methylation status at the CpG site of *CA9* promoter (Fig. 6C, D). Treatment of 5-Azacytidine (5-Aza), a specific inhibitor of DNA methylation, resulted in decreased DNA methylation of *CA9* and a significant induction of *CA9* mRNA level in TSCC15 cells (Fig. 6D, E).

Next, to further investigate whether DNMT1 is the yet-to-be found factor for *CA9* methylation, we tested the effects of DNMT1 on *CA9*. By using two independent siRNAs specifically targeting DNMT1, we found that DNMT1 knockdown significantly increased the expression level of CA9 in TSCC15 cells (Fig. 6F, G). Moreover, knockdown of DNMT1 greatly enhanced the TSCC15 cells’ migration rate in a CA9-dependent manner (Fig. 6H–K). Consistently with these results, the ChIP assay showed a hypoxia-induced recruitment of DNMT1 specifically to the CpG dinucleotides containing region located on the *CA9* promoter (Fig. 6L). The above results indicated that a DNMT1-mediated DNA methylation event occurs in the presence of hypoxia to negatively regulate the expression level of CA9.

To detect whether *887S* regulates *CA9* through DNMT1-meidiated DNA methylation, we first tested whether *887S* would affect the DNA methylation and the recruitment of DNMT1 on *CA9*. As expected, we found DNA methylation of *CA9* were dramatically increased upon *887S* overexpression (Fig. 6M, N). The DNMT1 ChIP assay also indicated a positive answer because that the recruitment of DNMT1 to the CpG dinucleotides of *CA9* promoter was abolished in *887S* knockout cells (Fig. 6O). By using the similar ChIRP assay strategy as that of *887L*, we found that *887S* exhibited a hypoxia-enhanced interaction with the CpG site (Fig. 6P, Q). These results strongly suggested that the DNA methylation of *CA9* and recruitment of DNMT1 to the *CA9* promoter are dependent on the action of *887S*.

Next, we wanted to investigate whether the *887S*-mediated CA9 inhibition is through DNMT1-mediated DNA methylation. We treated the *887S*-overexpressed TSCC15 cells with siDNMT1 or 5-Aza and found that *887S* lost its inhibitory ability on the expression of CA9 when DNMT1 was knockdown or DNA methylation process was blocked (Fig. 6R–T). Collectively, our results suggested that the hypoxia-induced *887S* mediates a DNMT1-dependent inhibitory effect on CA9 expression through DNA methylation in the presence of hypoxia.

### The interaction between lncRNA*-887S* and *887L* is regulated by the concentration of oxygen

**Fig. 7:**
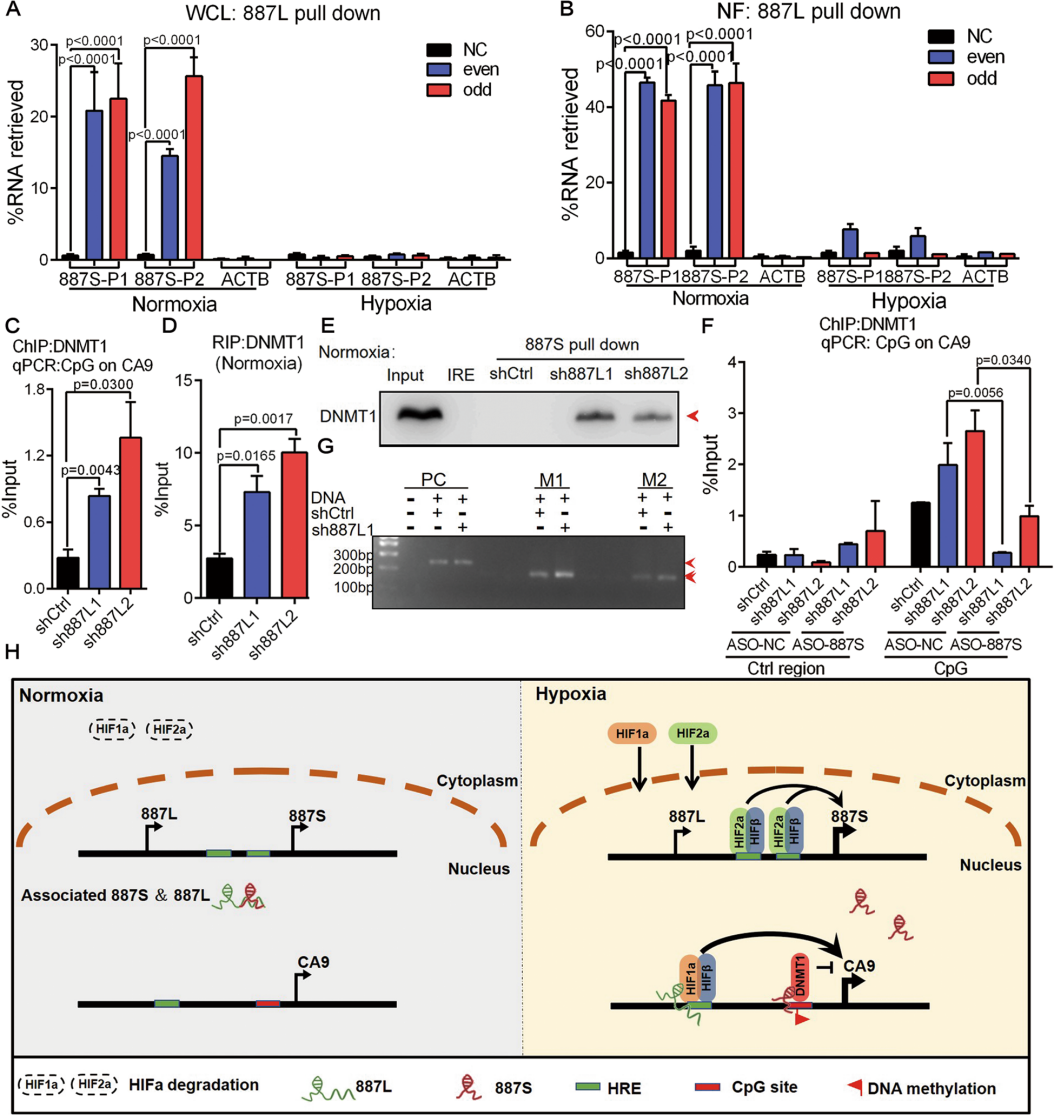
*887L* interacts with *887S* under normoxia and exhibits an inhibitory effect on *887S*. **A** *887S* was captured in the *887L*-precipitated complex under normoxia, instead of hypoxia. *887S*-P1 and *887S*-P2: primer sets for *887S* detection (*n*=2). WCL: Whole cell lysate. **B** *887S* was captured in the *887L*-precipitated complex under normoxia, instead of hypoxia. *887S*-P1 and *887S*-P2: primer sets for *887S* detection (*n*=2). NF: Nuclear fraction. **C** DNMT1 ChIP assay showed the changes of DNMT1 recruitment to *CA9*’s CpG site in *887L* knockdown cells under normoxia (*n*=3). **D** DNMT1 RIP assay showed the interaction of the DNMT1 and *887S* in *887L* knockdown cells under normoxia (*n*=3). **E** Representative image of RNA pull-down assay showed the association of DNMT1 and *887S* in *887L* knockdown cells under normoxia (*n*=3). IRE: a control RNA provided by Pierce RNA 3′ End Desthiobiotinylation kit. **F** Silencing of *887S* RNA by antisense oligonucleotide (ASO) (ASO-*887S*) abolished the recruitment of DNMT1 to the CpG site of *CA9* in *887L* knockdown cells under normoxia, as determined by ChIP-qPCR assay (*n*=3). **G** Representative image of methylation status of the CpG sites on CA9 promoter in sh*887L*1 cells and control cells. PC: a primer set to detect the C to T conversion which is provided by the MSP kit. **H** A schematic illustration of the *CA9* transcriptional regulation by interplay between *887S-* and *887L*-mediated pathways. Data are shown as means ± SEMs. *P* values are calculated using Student’s *t* test

The observation that *887L*-mediated HIF1α and *887S*-mediated DNMT1 recruitments were both hypoxia-induced strongly suggested that dynamic change of oxygen level acts as a critical regulatory signal for *887S* and *887L*. It was interesting to further investigate the underlying mechanism of the hypoxia-induced regulation of *887S* and *887L*. The fractionationing assay showed that the majority of *887S* and *887L* were DNA-bound under hypoxia (Fig. S4B). Although some DNA-bound *887S* and *887L* could be detected under normoxia, the DNA-bound fractions for both RNAs had been dramatically decreased (Fig. S4B). By using the *887L* antisense probes shown in Fig. 5F, G, we performed RNA antisense purification assay and RT-qPCR to investigate the potential interaction between *887S* and *887L*. These experiments were conducted using either whole cell lysates (Fig. 7A) or nuclear fractions (Fig. 7B). Our results detected an interaction between *887S* and *887L* exclusively under normoxia in both whole cell lysates and nuclear fractions (Fig. 7A, B). In addition, our results showed that *887S* and *887L* did not associate with their cis-acting elements under normoxia (Fig. 5G, and 6Q). Together, our results indicated that the interaction status between *887S* and *887L* is dynamically regulated by the concentration of oxygen. The interaction between *887S* and *887L* is likely to occur in the nucleoplasm.

In Fig. 5H, we have provided evidence that modulation of *887S* expression level had no effect on HIF1α recruitment to *CA9* promoter, suggesting that *887S* is not an inhibitory factor to *887L-*HIF1α axis. However, it did not exclude the possibility that *887L* acted as a regulatory factor for *887S*. To test our hypothesis, we knocked down *887L* and performed a series of investigations. Our data showed that the recruitment of DNMT1 to the *CA9* promoter was increased in both sh*887L*1 and sh*887L*2 cell lines (Fig. 7C). In addition, *887L* knockdown dramatically increased the association of DNMT1:*887S*, observed by both DNMT1 RIP assay (Fig. 7D) and *887S* RNA pull-down assay (Fig. 7E).

In order to investigate whether the observed DNMT1 recruitment in *887L* knockdown lines shown in Fig. 7C was mediated through *887S*, we knocked down *887S* in the two sh*887L* lines and performed DNMT1 ChIP assay. Our data showed that DNMT1 recruitment was completely abolished after *887S* knockdown (Fig. 7F). Moreover, we also observed a concomitant increase of DNA methylation in tested CpG region at *CA9* promoter after *887L* knockdown (Fig. 7G). All together, our data strongly indicated that the interaction between *887S* and *887L* is dependent on the oxygen concentration. In addition, *887L* exhibits a one-directional inhibitory effect on *887S*, which provides an indirect regulatory mechanism that explains the role of *887L* on *CA9* under normoxia (Fig. S12).

## Discussion

In this work, we report the roles of two newly identified lncRNA variants in TSCC and the molecular mechanisms of how the activity of the oncoprotein CA9 is precisely maintained by these two antagonistic but interplaying lncRNA variants (Fig. 7H).

CA9 plays an essential role in hypoxia adaptation and oncogenic progression [8, 53, 63, 64]. However, the upstream regulatory mechanism of CA9 is largely unknown. In this work, the two investigated lncRNA variants, *887S* and *887L*, are both upregulated in TSCCs but function in opposite directions on *CA9* transcription. Through recruiting HIF1α, *887L* promotes *CA9*’s expression while *887S* inhibits *CA9* through DNMT1-DNA methylation under hypoxia.

CA9 is well known for being upregulated by hypoxia [56–58]. Surprisingly but intriguingly, both activation signals (*887L*-HIF1α) and repressive signals (*887S*-DNMT1-DNA methylation) to CA9 are observed under the same condition-hypoxia. Eukaryotic cells have developed sophisticated mechanisms to establish fine-controlled balance between activation and repression signals in a broad range of cellular events [65–68]. Chronic reduction of HIFα proteins has been observed in tumor cells after prolonged hypoxia treatment [11, 12]. It has been proposed that such chronic reduction of HIFα can protect tumor cells from unwanted deleterious consequences [11, 12]. We speculate that *887S*-dependent inhibitory action is part of the fine control of CA9 expression to keep CA9 from being over-activated under hypoxia. As a result of this, the expression level of the inhibitory *887S* should be proportional to that of *887L*, the positive regulator of CA9. Supporting this, we consistently observed a synchronous expression pattern of *887S* and *887L* in the tumor cells (Fig. S3A, B) and the in-house collected TSCC patient specimens (Fig. S3C). Another interesting observation is that *887S* is enhanced by HIF2α, instead of HIF1α upon hypoxia. It has been recently reported that HIF1α and HIF2α have unique and sometimes opposing target genes [52, 69]. Together, our results indicate that the expression level of *CA9* is under fine and sophisticated control at the transcriptional level through an lncRNA-dependent manner.

Both HRE and DNA methylation sites are broadly distributed across human genome. It would be reasonable to speculate that the two investigated lncRNA variants may affect other targets through HIF1α and DNMT1 to reconcile the RNAs’ functions in tumor progression.

Enormous quantity in the human genome and high sensitivity to the environment change endow lncRNA transcripts as perspective regulators for human diseases [15–18, 70]. We [71] and other independent groups [72–74] have found that lncRNAs use AP, AS, and alternative polyadenylation (APA) as general strategies to further expand the genome diversity. Although several studies have been reported [75, 76], the biological significance of lncRNA variants generated by these upstream biogenesis signals are largely unknown. In this work, we found that the interaction between *887S* and *887L*, a pair of lncRNA variants, is dynamic and regulated by the oxygen concentration. In the presence of higher oxygen concentration, *887S* and *887L* interact. When the oxygen level falls, the two lncRNAs are separated from each other. Each individual transcript “talks” with their responsive DNA cis-acting elements on *CA9*. It would be of great interest to investigate how the dynamic interaction between these two RNAs is regulated in the future. LncRNAs are highly sensitive to their cellular environment [77]. It is very likely that certain hypoxia-triggered microenvironment alterations (e.g., pH) induce the signal-specific higher structural changes of either RNA variant or both to modulate their RNA-RNA interaction status, each RNA’s binding ability to the corresponding cis-acting element and thus their regulation on CA9 expression.

Growing clinical evidence has suggested that CA9 can serve as a prognostic biomarker and a therapeutic target [8, 53, 63, 64]. To date, about 20 CA inhibitors (CAIs) have been underwent clinical trials for anti-cancer therapies [78]. However, due to the similarity among CA family members in sequence, structure, and particularly within their active sites, it is difficult to design the isoform-specific CA inhibitors [54, 58, 79]. The specific effects of *887S* and *887L* on CA9 among CA family members indicate that targeting upstream molecules may offer a suitable alternative option for CA9-based anti-cancer drug design.

Together, our work provides a hypoxia-permitted “talk” between the two investigated lncRNA variants to their cis-acting elements and thus fulfill their direct regulation on *CA9* transcription. Our findings bring up a novel lncRNA-mediated mechanism that lncRNAs act as environmental sensors and guide the appropriate cellular adaptation through precisely controlled transcription network.

## Conclusion

In summary, we report the squamous cell carcinoma highly associated genomic locus Chr3q29 can generate a pair of alternative promoter-regulated lncRNA variants *887S* and *887L*. The two lncRNAs are differentially responded to hypoxia and oppositely control tumorigenesis through distinct but interplaying transcriptional regulatory axis on oncogene *CA9*. This integrated and coordinated collaboration between *887S* and *887L* results in a fine controlled expression level of CA9. Our work expands the current understanding on tumor hypoxia adaptation and provides a promising new therapeutic strategy in anti-cancer treatment.

## Methods

### Cell culture and treatment

All cell lines were purchased from the American Type Culture Collection (ATCC). The HEK 293T, Hela cells were cultured in Dulbecco’s modified Eagle’s medium (DMEM) (Gibco, USA). MCF-7, MDA-MB-231, SK-BR3, and HCT116 cells were cultured in RPMI1640 medium (Gibco, USA). TSCC9, TSCC15, TSCC25, and SH-SY5Y cells were cultured in Dulbecco’s modified Eagle’s medium/Nutrient Mixture F-12 (DMEM/F-12) medium (Sigma, USA), containing 10% fetal bovine serum (FBS, Gibco, USA) and 1% penicillin-streptomycin (WISENT Inc., CA). MCF-10A cells were cultured in DMEM/F-12 medium supplied with 5% horse serum (Gibco, USA), 10μg/ml insulin (Roche, USA), 20ng/ml EGF2 (Sigma-Aldrich, USA), 100ng/ml cholera toxin (Sigma, USA), 500ng/ml hydrocortisone (Melonepharma, CHINA), and 1% penicillin-streptomycin. All cells were cultured under humidified atmosphere of 5% CO_2_ at 37°C. For the 5-Azacytidine treatment, 1μM of 5-Azacytidine (5-Aza, MedChem Express) was added into culture medium when cell density was around 50%. After 48h treatment with 5-Aza, TSCC15 cells were collected and extracted for further analysis. For the hypoxia treatment, cells were cultured under 1% O_2_ at hypoxia station (Don Whitley Scientific:H35 hypoxystation, UK).

### Xenograft mouse model

All animal studies were conducted with the approval from the Animal Research Ethics Committee of the University of Science and Technology of China (Approval number USTCACUC-1801020). Female BALB/C nude mice at the age of 5 to 6 weeks (Nanjing Biomedical Research Institute of Nanjing University) were used for model construction. Briefly, differently treated tumor cells were trypsinized and harvested, then 1x10^7^ TSCC15 or 3x10^6^ TSCC25 cells in serum-free medium containing 20% matrigel with a total volume of 0.2ml were injected subcutaneously to the inguina of the mice. Four weeks later, the mice were sacrificed by cervical dislocation and noticeable tumors were immediately excised. The tumor volumes were measured with a caliper and calculated using the equation *V*=0.5×*L*×*W*2, where *V* is the volume, *L* is the length (longest dimension), and *W* is the width (shortest dimension).

### Clinical samples

TSCC specimens including paired paracancerous tissues and tumor samples were collected from the Department of Oral and Maxillofacial Surgery, Peking University School and Hospital of Stomatology. The research protocol was approved by the Ethics Committees for Human Experiments of Peking University School and Hospital of Stomatology. All participants signed and informed consent prior to sample collection (Approval number PKUSSIRB-2013009).

### RNA-seq data resources and analysis

RNA expression data (RNA-seq) of The Cancer Genome Atlas (TCGA; http://cancergenome.nih.gov, RNA-seq Version 2) was downloaded. After excluding the cancer type with less than 10 normal tissue samples, we got 5540 samples in total, comprising 4907 primary solid tumor samples and 631 normal samples from 13 cancer types**.** The differentially expressed lncRNAs between tumor and normal samples were screened by performing cross-value association analysis (CVAA [21]). Briefly, the downloaded RNA-seq data sets were denoted as the matrix E with *m* rows (samples) and *n* columns (lncRNAs). Looping comparison was conducted as described before [21]. According to the log-linear model theory, Likelihood ratio test (LRT) value can statistically represent the significance score of genes which are differently expressed between samples [21]. And Logarithm 2 of odds ratio (LOD) values can indicate the overall change direction of a certain gene (upregulation: LOD > 0, downregulation: LOD < 0) [21]. Therefore, the lncRNAs running for looping comparison were scored and ranked according to LRT in descending order. The downregulated or upregulated lncRNAs were sorted by LOD. Next, to identify the hypoxia-regulated lncRNAs from the resulted top 50 CVAA lncRNAs, we searched the published reports on PubMed and found 6 CVAA lncRNAs whose expression levels were regulated by hypoxia treatment [22–31]. These 6 CVAA lncRNAs included *CDKN2B-AS1*, *PVT1*, *HOTAIR*, *UCA1*, *MIR31HG*, and *LINC00887* and were listed as hypoxia-regulated CVAA lncRNAs in Supplementary Table 1.

### Partial correlation analyses (PCA)

PCA was conducted as previously described [80]. Briefly, RNA-seq data-sets retrieved from TCGA was downloaded and then was mathematically calculated by the R (v 3.0.2) package “ggm” (v2.3) with the function “pcor” for given sets of TCGA expression data. The “pcor” function is to analyzed pair-wise gene expression correlation. With the cutoff of |cors| > 0.3, 634 genes showed to be *LINC00887* co-expressed and the top correlated genes were listed in Fig. 1C.

### Gene ontology (GO) analyses

The PCA generated *LINC00887-*correlated genes were applied to Database Annotation Visualization and Integrated Discovery (DAVID) (v6.8) (http://david.abcc.ncifcrf.gov/) for gene ontology (GO) enrichment analyses. The top 10 terms from the resulted significant GO terms (*p* < 0.01) were shown in Fig. 1B.

### RNA isolation and reverse transcription (RT)—quantitative PCR (qPCR)

Total RNA was isolated using Trizol Reagent (Ambion, USA) according to the manufacturer’s instructions and treated with RNase-free DNase I (Thermo Scientific, USA). One Drop® OD-1000 Spectrophotometer (Nanjing Wuyi Corporation, CHINA) was used to measure RNA concentration and purity. Reverse-transcription was performed using HiScript II One Step RT-PCR Kit (Vazyme, CHINA) according to the manufacturer’s instructions. Reaction without transcriptase was performed as a no-RT control. Real-time PCR was performed using SYBR® Green Master Mix (Vazyme, CHINA) according to the manufacturer’s instructions on Light Cycle® 96 (Roche, USA). GAPDH RNA was used as internal control for Figure S1A, and 18S RNA was used as internal control for all the other RT-qPCRs. The primers used for the real-time PCR are listed in Supplementary Table S2. Fold changes were determined using the relative quantification 2^-△△CT^ method.

### Western blot

Cells were lysed in RIPA buffer (Vazyme, CHINA) for 45min on ice and centrifuged at 2500g for 5min at 4°C. After quantification using One Drop® OD-1000 Spectrophotometer (Nanjing Wuyi Corporation, CHINA), whole cell lysates were separated by SDS-PAGE under denaturing conditions and transferred to PVDF membranes (Millipore, USA). The membranes were blocked in 5% BSA (Sangon, CHINA) and then incubated with primary antibodies for β-ACTIN (Proteintech, USA), β-TUBULIN (Proteintech, USA), CA9 (Absolute Antibody, UK), FBL (Proteintech, USA), HIF1α (Novus, USA), HIF2α (BD, USA), and Histone H2B (Abcam, UK). The PVDF membranes were then incubated with secondary antibodies conjugated with horseradish peroxidase (Proteintech, USA). Immunoreactive proteins were visualized using the SuperSignal® West Femto Maximum Sensitivity Substrate (Thermo Scientific, USA) on ChemiDoc-It (UVP, UK).

### Rapid amplification of cDNA ends (RACE)

RACE was performed with the 5′RACE System and 3′RACE System Kit (Invitrogen, USA) according to the manufacturer’s instructions. Briefly, the 5′RACE system is a set of prequalified reagents intended for synthesis of the first-strand cDNA, purification of the first-strand cDNA, homopolymeric tailing, and preparation of target cDNA for subsequent amplification by PCR. The 3′RACE procedure is also summarized as cDNA synthesis, RNA template degraded with RNase H, and amplificated by PCR. The primers used are as follows (GSP: Gene specific Primer):

5’RACE: cDNA GSP: TAAACAGGTGAAACACT;

PCR GSP: ACATTCGCAAGAGGGTGACAGT;

Nest PCR GSP: CGTCCCCAGGGCACCAGAATA;

3’RACE: GSP1: CAGGACCAGCCAGCCCTTTC;

GSP2: ATGGGAGCCTGGCCTTTGAG;

Primer-887 (1):

F: CACAGCAGCCTCCTCTTAAAC; R: CTTTTCTCTCCCATGCTGAGC

Primer-887 (2):

F: GGCCTTTGAGATTCCTGCGA; R: ATGCCTCAGTCGAAGGGAGA

Primer-887S: F: CTGCTCAGACACCGTTGC; R: GATGTGGTCTCACTCTGTTGC;

Primer-887L: F: CTGCTCAGACACCGTTGC; R: CTTGATGCTTTTACAGGCTCTC

### siRNA and ASO oligonucleotides

siRNA and ASO oligos were designed and synthesized from Ribobio (Guangzhou, China). In each case, cells were plated at about 40% confluence before transfection. 20μM oligos were used for transient transfections into cells with Lipofectamine 3000 (Invitrogen) for 48h. The knockdown efficiency was validated with RT-qPCR. The sequences of siRNA and ASO are listed in Supplementary Table S3.

### Plasmids construction for luciferase assay

The promoter of *887S* (924bp) containing two hypoxic response elements (HREs) was amplified by PCR with the template of TSCC15 cell genomic DNA. Primers with the following restriction sites were used for *887S* promoter: forward, 5′-CCCTCGAGGGCAAGTTTCCCTACTGCCTCC-3′ (XhoI); reverse, 5′-CCAAGCTTGGATTCTGTTTCTCATCAGGCG-3′ (HindIII). The clones were ligated into PGL3-basic vector’s upstream of the luciferase gene to construct the wild type *887S* promoter reporter. The HRE sites in wild type *887S* promoter reporter were mutated from 5′-CGTG-3′ to 5′-ATAA-3′ respectively by overlap extension PCR to generate mutant *887S* promoter reporter. Similar, the promoter of *CA9* (2024bp) was amplified using PCR with the template of TSCC15 cell genomic DNA. Primers with the following restriction sites were used for *CA9* promoter: forward, 5′-CCGCTCGAGCGGAGTTCTGCATCAACCTGGTT-3′ (XhoI); reverse, 5′-CCCAAGCTTGGGTGTACGTGCATTGGAAACG-3′ (HindIII). The clones were ligated into PGL3-basic vector upstream of luciferase gene to construct the *CA9* promoter reporter. The sequences of all plasmids were validated before use.

### Dual Luciferase Reporter Assay

Cells were pre-seeded in a 24-well plate at a density of 1×10^5^ cells/dish. On the following day, the cells were co-transfected with 1μg of constructed promoter reporter plasmids or control plasmids and 200ng of pRL-TK plasmid by Lipofectamine3000 (Invitrogen, USA). Twenty-four hours after transfection, cells were collected. Firefly and Renilla luciferase activities were sequentially measured by a Dual-LuciferaseTM Reporter Assay system (Promega). Luciferase activity was normalized by Renilla activity for each well. All assays and analyses were carried out in triplicate.

### shRNA lentiviral transfection and stable cell lines establishment

Two independent shRNAs specifically targeting two different regions of *887L* were designed and separately cloned into pLKO.1 vector to generate *887L* shRNAs. *887L* shRNAs and packaging vectors (pREV, pGag/pol, pVSVG) were co-transfected into HEK 293T cells using the lipofectamine 2000 (Invitrogen, USA). The medium was changed 6h after transfection with 20% FBS DMEM medium, and the supernatant containing lentivirus was collected 48h after transfection. Meanwhile, TSCC15 cells growing in the presence of 8μg/ml polybrene (Sigma, USA) were prepared to reach a confluency of 70–80% for viral infection. Stable monoclonal infectants were generated under the treatment of 1μg/ml puromycin (Gibco, USA), and monoclonal infectants with *887L* knocking down efficiency more than 50% were selected for further tests. The shRNA sequences are as follows:

shRNA1:

F: ccggCTCACGTTGTCACCTTAGTggatccACTAAGGTGACAACGTGAGtttttg;

R: attcaaaaaCTCACGTTGTCACCTTAGTggatccACTAAGGTGACAACGTGAG;

shRNA2:

F: ccggCACTTCTCGTCACCACTA TggatccATAGTGGTGACGAGAAGTGtttttg;

R:aattcaaaaaCACTTCTCGTCACCACTATggatccATAGTGGTGACGAGAAGTG.

### Plasmid overexpression and stable cell line establishment

*887S* or *887L* was amplified using PCR with Phanta^TM^ Super-Fidelity DNA Polymerase (Vazyme, CHINA) separately and subsequently cloned into the pcDNA3.1(+) (Invitrogen, USA) overexpression plasmid. Stable cell lines were constructed after transfection with the pcDNA3.1-*887S* or pcDNA3.1-*887L* plasmid and grew in the presence of 200 μg/ml G418 (WISENT Inc., CA). Samples with more than 2 times overexpression rate were selected for further tests. The PCR primers were used as follows:

*887S*:F: AGGAATTCCTGCTCAGACACCGTTGC;

*887S*:R: TAGGTACCGATGTGGTCTCACTCTGTTGC;

*887L*:F: AGGAATTCCTGCTCAGACACCGTTGC;

*887L*:R: TAGGTACCCTTGATGCTTTTACAGGCTCTC.

### CRISPR-Cas9 and gRNA lentiviral transfection

All lentiviral vectors, including the CRISPR-Cas9 vector and guide RNA vector, were supplied by Cyagen Bioscience Corporation, USA (Service Agreement Number: MBS161230JX1). The experiment was performed according to the manufacturers’ instructions. Two independent guide RNAs sequences are sgRNA1: 5′-GTGAGCTGCAGAGGTAGCCG-3′; sgRNA2: 5′-AGCACGTGCGCTTGCTCTGC-3′. The resulted *887S* knockout cell lines (HREmut1 and HREmut2) were further used in desired experiments under hypoxia.

### Colony formation assay

200 or 400 cells collected during the logarithmic growth phase were seeded into 6-well cell culture plates (Corning, USA). The medium was replaced every 5 days. Cells were fixed with methanol and stained with 0.1% or 2.5% crystal violet after 7 or 14 days, then washed with 1×PBS. Colonies containing over 50 cells were counted manually (200×) under a microscope (Olympus, JAPAN). The colony formation rate was calculated as the percentage of colonies per numbers of inoculated cells (The colony formation rate (%) = (colonies per numbers / 200) ×100%).

### Transwell assay

Transwell chambers inserting with an 8-μm pore size in 24-well cell culture plates (Corning, USA) were used. 1×10^5^ or 5×10^4^ of cells were suspended in 100ul serum-free media and added to the upper chamber. Complete medium containing 20% fetal bovine serum (0.7ml) was added to the bottom chamber as a chemo-attractant. The chambers were incubated in cell culture incubator for 24h. After incubation, the non-migrated cells in the upper chamber were removed with cotton swabs. The membranes were fixed with 4% paraformaldehyde (Sangon, USA) and stained with 0.1% crystal violet, and then cells from seven random fields (10×) were counted using light microscopy (Olympus, JAPAN). The relative mobility (‰) = (total cells of seven fields × 22.763)/(7 × initial incubated cells).

### Intracellular and extracellular pH detection

BCECF-AM kit (Invitrogen, USA) was used to measure intracellular pH. BCECF-AM is a fluorescence probe sensitive to intracellular pH, which penetrates the cell membrane and is hydrolyzed into BCECF by esterase after entering the cell. BCECF can be excited to produce green fluorescence under the appropriate pH value [81]. Cell suspensions were prepared with HEPES at a concentration of 3×10^6^ cells/ml. A final concentration of BCECF-AM at 3μM was obtained by adding 1 mM BCECF-AM/DMSO solution to the cell suspension (1/300 volume of the cell suspension). After 30min of incubation at 37°C, the cells were washed with HEPES buffer 3 times and adjust to 3×10^6^ cells/ml. Finally, the fluorescence intensity of the cells was measured by fluorescence microscopy (Olympus, JAPAN). Detection of the extracellular pH value has been described previously [82, 83]. Briefly, the media from cultured cell were collected and detected by benchtop pH meter (Mettler Toledo, Switzerland).

### Hematoxylin and eosin stain

The xenograft tumors were fixed with 4% paraformaldehyde, embedded in paraffin, and cut into 5μm sections. Then, the sections were transferred to adhesive-coated slides. All the sections were routinely deparaffinized and rehydrated, followed by incubation of hematoxylin (Sangon Biotech) for 8min at room temperature. After being rinsed with running water, the sections were treated by 1% ethanol hydrochloride to remove the excessive binding of hematoxylin dye and the adsorbed hematoxylin dye in cytoplasm. Next, the sections were respectively rinsed in running water and distilled water, then immersed in eosin dyes (Sangon Biotech) for 1min at room temperature. After the process of dehydration and fresh xylene treatment, the slides were examined and photographed by light microscopy (Olympus, JAPAN).

### Cytosolic/nuclear fractionation

Cytosolic/nuclear fractionation assay was performed as previously described [84]. Briefly, TSCC15 cells were lysed in RSB-100 buffer (100mM Tris-HCl, 100mM NaCl, 2.5mM MaCl_2_, 40μg/ml digitonin). The supernatant fraction was collected as cytosolic fraction after centrifugation. The nuclear part was collected with supernatant after resuspended cell pellet in RSB-100T (100mM Tris-HCl, 100mM NaCl, 2.5mM MaCl_2_, 40μg/ml digitonin, 0.5% TritonX-100). After sonication, the soluble DNA-bound RNA fraction was collected.

### Fluorescence in situ hybridization analysis (FISH)

Cy3-labeled FISH probes for *887L*, β-ACTIN (ACTB), and Cy5 labeled FISH probes for *887S*, β-ACTIN (ACTB) RNAs were designed and synthesized by GenePharma (Shanghai, China). The FISH assay was conducted according to the instruction of Fluorescent In Situ Hybridization Kit (GenePharma, Shanghai, China). Briefly, cells growing on glass coverslips were fixed with ice-cold 4% paraformaldehyde for 15 min and blocked with pre-hybridization buffer for 30min. The desired RNA probes (4μM) were incubated with hybridization solution for 5min at 73°C before hybridization. The cells were then incubated with the RNA probes (4μM) in the dark at 37°C for 12~16h, followed by three times of wash with 4×SSC containing 0.1% Tween-20. Finally, the cell nuclei were stained with Hochst33342 (Thermo, America). Images were taken by confocal fluorescence microscope (FV1200MPE-share, Olympus, Japan).

### In vitro transcription and RNA pull-down

PCR was used to evaluate *887S*, with the following primers that bound to the T7 promoter (*887S*: F: TAATACGACTCACTATAGGGCTCAGACACCGTTGC, R: GATGTGGTCTCACTCTGTTGC). The RNA products were transcribed by a T7 RNA polymerase kit (Invitrogen, USA) in vitro, treated with RNase-free TURBO DNase I (Invitrogen, USA), and labeled with the Pierce RNA 3′ End Desthiobiotinylation Kit (Thermo Scientific, USA). Cells were lysed in lysis buffer (50mM Tris-Cl, pH 7.0, 10 mM EDTA, 1% SDS, protease and phosphatase inhibitor cocktail, RNase inhibitor) on ice for 1–2h. At the same time, 3′ biotin-labeled RNA and magnetic beads were incubated for 30min at room temperature. Subsequently, the cell supernatants were collected after centrifugation at 13,000g for 10min at 4°C and added to the 3′ biotin-labelled RNA-magnetic beads mixture rotating for 2h at 4°C. After washing the beads for four times, the *887S*-interacting proteins were subsequently identified by western blot.

### RNA immunoprecipitation (RIP)

A total of 1×10^7^ cells were harvested and suspended in 10 ml of PBS with 1% formaldehyde to fix for 10min at room temperature. Cross-linking was stopped by adding glycine to a final concentration of 0.25M, followed by incubation at room temperature for 5min. After pelleting cells at 1000rpm for 5min, cell pellet was isolated and lysed with 1ml of RIPA buffer (Vayzme, China) supplemented with RNase inhibitor (Vayzme, China) followed by sonication (6×, 6 s each time). After centrifuged at 14,000rpm for 10min at 4°C, the supernatant was pre-cleared with 25μl of Dynabeads Protein A/G at 4°C for 1h. Then, RNA-protein complexes were enriched by the beads conjugated with anti-HIF1α (Novus, USA; BD, USA), anti-DNMT1 (Abcam, UK), or IgG antibody at 4°C overnight. The RNA in beads complexes was isolated by TRIzol (Ambion, USA) and used to synthesize cDNA with SuperScript III Reverse Transcriptase (Vazyme, China), followed by RT-qPCR analysis.

### Chromatin immunoprecipitation assay (ChIP)

ChIP assay was performed as previously described [84]. Briefly, TSCC15 cells were cross-linked with 1% formaldehyde for 10min. ChIP assays were performed using anti-HIF1α (Novus, USA; BD, USA) and anti-DNMT1 (Abcam, UK). Anti-IgG was used as a negative control. The DNA fragments were extracted and subjected to qPCR by primers detecting for corresponding cis-acting elements and negative control regions.

### Chromatin isolation by RNA purification (ChIRP)

For each ChIRP experiment, 2×10^8^ cells (293T or TSCC15) were used. The cells were cross-linked with formaldehyde and sonicated as previously described. Biotinylated RNA oligonucleotides of *887S* or *887L* were then hybridized with the cell lysates. The precipitated RNA or DNA was extracted and conducted for PCR by primers detecting for the desired regions and control regions. 293T is a cell line with no *887S* or *887L* expression and serves as a negative control. The sequences of probes are listed in Supplementary Table S3.

### DNA methylation detection

The methylation status of the CpG site within CA9 promoter was determined by methylation-specific PCR (MSP) as reported before [85]. A total of 1μg of genomic DNA from TSCC15 cells was treated with sodium bisulfite using the bisulfite conversion kit (Active Motif, USA). A pair of positive control conversion-specific PCR primer was used to assess the success of the bisulfite conversion according to the manufacturer’s instructions. The design of specific primers targeting the bisulfite-modified methylated CpG site of CA9 promoter was done by Methprimer software (University of California, San Francisco, CA). The primer sequences used to detect methylated CA9 promoter are listed below. The presence of the respective PCR products was shown by Gel Red staining after electrophoresis in 2% agarose gels. In addition, a negative control reaction without template DNA was done together with each PCR experiment.

M primer1-F: TTTAAGTTGAGTTTATGGTTTCGA;

M primer1-R: AAAAAACAAACTAACTCACAAAACG;

M primer2-F: TTTAAGTTGAGTTTATGGTTTCGA;

M primer2-R: AAAAACAAACTAACTCACAAAACG.

### Northern blot

RNAs from 293T, TSCC15, or TSCC25 cells were extracted with Trizol. Five microgram of RNA was separated on 1% formaldehyde agarose gel and blotted with biotin-labeled probes (General Biol, China). In vitro transcribed full length *887S* and *887L* RNA were used as positive controls and position markers. 293T is an *887S*/*887L* non-expression cell line and serves as a negative control. The probes used for Northern blot were list in Supplementary table S2.

### Quantification and statistical analysis

Each experiment was repeated independently at least three times, except for the ChIRP experiments (Fig. 5F, G, 6P, Q, and 7A, B) and the fractionationing assay (Fig. S4B) which were performed by two biological repeats. Statistical analysis was carried out using Microsoft Excel software and GraphPad Prism to assess the differences between experimental groups. Statistical significance was analyzed by two-tailed Student’s *t* test and expressed as a *P* value. *P*<0.05 were considered to be statistical significance.

## Supplementary Information

**Additional File 1: Supplementary Table1:** Top 50 CVAA lncRNAs with hypoxia regulated information. **Supplementary Table2:** Primer sequence. **Supplementary Table3:** siRNA, ASO, ChIRP, FISH and Northern blot probe sequence

**Additional File 2: Fig S1.** Up-regulation of *LINC00887* is correlated with poor survival rate of TSCC patients. Fig S2. The variety, existence and relative abundance of *887S*, *887L* and other *LINC00887* variants. **Fig S3.** The expression pattern of *887S* and *887L* in the indicated cell lines. **Fig S4.** *887S* and *887L* subcellular localization and coding potential. **Fig S5.** Efficiency of gain-of-function or loss-of-function experiments in the indicated *887S* and *887L* knockdown or overexpression cells. **Fig S6.** CA9 promotes tumor progression in TSCC. **Fig S7.** Expression level of CA9 in the indicated *887S* or *887L* modulated cells. **Fig S8.** *887S* and *887L* regulates intracellular pH in TSCC. **Fig S9.** Effects of *887S* and *887L* on xenograft growth in Balb/c (nu/nu) mice. **Fig S10.** Effects of ASO-mediated *887S* knockdown. **Fig S11.** In vitro transcribed *887S* visualized by agarose gel with 1% formaldehyde. **Fig S12.** *887L* promotes TSCC progression under normoxia.

**Additional File 3.** Raw blots and gel datasets

**Additional File 4.** The accession and information of TCGA-released RNA-seq datasets analysed in this paper

## Data Availability

All data generated or analyzed during this study are included in this published article, its supplementary information files and publicly available repositories. The RNA-seq datasets that support the findings of this study are available in TCGA (https://confluence.broadinstitute.org/display/GDAC/Home) and listed in Additional File 4. The analytic codes and procedures that support the findings of this study have been deposited in GitHub with the identifier (https://github.com/liqg/CVAA) [21].
