# Supplementary material for: A pair of long intergenic non-coding RNA LINC00887 variants act antagonistically to control Carbonic Anhydrase IX transcription upon hypoxia in tongue squamous carcinoma progression
Source: BMC Biol. 2021 Sep 7;19:192. doi: 10.1186/s12915-021-01112-2 (PMC8422755; doi:10.1186/s12915-021-01112-2)
Supplement: Supplementary file 1 — Additional File 1: Supplementary Table1: Top 50 CVAA lncRNAs with hypoxia regulated information. Supplementary Table2: Primer sequence. Supplementary Table3: siRNA, ASO, ChIRP, FISH and Northern blot probe sequence [file 12915_2021_1112_MOESM1_ESM.docx]

**Supplementary Table1: Top 50 CVAA lncRNAs with hypoxia regulated information**

| **Name** | **LRT** | **LOD** | **Hypoxia induced lncRNA** | **Reference** |
| --- | --- | --- | --- | --- |
| PGM5-AS1 | 424384.3 | -1.69216 | N.A. | N.A. |
| MFSD4A-AS1 | 347026.4 | -1.12562 | N.A. | N.A. |
| PRDM16-DT | 338057.2 | -1.28914 | N.A. | N.A. |
| TRHDE-AS1 | 333418.5 | -1.36191 | N.A. | N.A. |
| CDKN2B-AS1 | 286004.3 | 0.764822 | Yes | (22, 23) |
| EPR1 | 284262.5 | 1.678758 | N.A. | N.A. |
| LINC00887 | 271388 | 0.899902 | Yes | (24) |
| LINC00634 | 268686.4 | 1.202307 | N.A. | N.A. |
| LINC00473 | 266659 | -0.95828 | N.A. | N.A. |
| GATA3-AS1 | 256805.3 | -0.28704 | N.A. | N.A. |
| LINC00461 | 252189.1 | -0.17646 | N.A. | N.A. |
| MESTIT1 | 245295.2 | -0.9725 | N.A. | N.A. |
| FAM167A-AS1 | 244114.9 | -1.39995 | N.A. | N.A. |
| LINC00602 | 243764.1 | -0.98644 | N.A. | N.A. |
| PART1 | 243474 | -0.4459 | N.A. | N.A. |
| NRAD1 | 236155.7 | -0.82462 | N.A. | N.A. |
| MGC27382 | 233863.5 | -1.21604 | N.A. | N.A. |
| WT1-AS | 233156.5 | 0.113476 | N.A. | N.A. |
| PVT1 | 227206.3 | 1.165496 | Yes | (25-28) |
| NKAIN3-IT1 | 224711.6 | -0.72708 | N.A. | N.A. |
| LINC00092 | 215029.6 | -0.9435 | N.A. | N.A. |
| LINC00922 | 214687.9 | 1.108018 | N.A. | N.A. |
| LHFPL3-AS2 | 214278.6 | -0.82066 | N.A. | N.A. |
| NPSR1-AS1 | 213425.8 | -0.07189 | N.A. | N.A. |
| HOXA11-AS | 213392.7 | 0.33842 | N.A. | N.A. |
| HAND2-AS1 | 213023.6 | -0.84386 | N.A. | N.A. |
| MIR924HG | 212875.1 | 0.969179 | N.A. | N.A. |
| FAM83A-AS1 | 210717.3 | 0.85363 | N.A. | N.A. |
| HOTAIR | 208245.2 | 0.955376 | Yes | (29) |
| LRRC52-AS1 | 206663.3 | 0.107149 | N.A. | N.A. |
| LINC01020 | 205129.2 | -2.84877 | N.A. | N.A. |
| MIR99AHG | 203666.6 | -1.12676 | N.A. | N.A. |
| DSCR8 | 203094.9 | 0.54974 | N.A. | N.A. |
| SILC1 | 202308.3 | -0.57853 | N.A. | N.A. |
| B3GALT5-AS1 | 201759.1 | -0.49749 | N.A. | N.A. |
| C1orf140 | 198685.4 | -0.99603 | N.A. | N.A. |
| FIRRE | 197961.8 | 0.86514 | N.A. | N.A. |
| LOC440173 | 195818.2 | -0.36386 | N.A. | N.A. |
| **Name** | **LRT** | **LOD** | **Hypoxia induced lncRNA** | **Reference** |
| SSTR5-AS1 | 195247.5 | -0.33666 | N.A. | N.A. |
| LINC00626 | 195050.7 | 0.866148 | N.A. | N.A. |
| DLX6-AS1 | 193748.9 | 0.933734 | N.A. | N.A. |
| C20orf166-AS1 | 193204.9 | -0.87675 | N.A. | N.A. |
| UCA1 | 191331.6 | 0.205079 | Yes | (30) |
| DIO3OS | 190885.9 | -0.81454 | N.A. | N.A. |
| LOC283856 | 189316.5 | -0.8744 | N.A. | N.A. |
| LINC00908 | 188649.4 | -0.7132 | N.A. | N.A. |
| LINK-A | 188135.6 | -0.49502 | No | (86, 87) |
| MIR31HG | 188042.7 | 0.265015 | Yes | (31) |
| TINCR | 185306.2 | 0.044928 | N.A. | N.A. |
| CCL15-CCL14 | 182649.7 | -0.96768 | N.A. | N.A. |

Note: CVAA: cross-value association analysis; LRT: Likelihood ratio test; LOD: Logarithm 2 of odds ratio; N.A.: Non Appliable; Yes: Reported hypoxia-inducible lncRNA. No: Reported non hypoxia-inducible lncRNA.

**Supplementary Table2: Primer sequence**

| **Name** | **Primer sequence** | |
| --- | --- | --- |
| GAPDH | Forward | ACATCATCCCTGCCTCTACG |
|  | Reverse | CCTGCTTCACCACCTTCTTG |
| 18S | Forward | GTAACCCGTTGAACCCCATT |
|  | Reverse | CCATCCAATCGGTAGTAGCG |
| HIF1α | Forward | GCTTTAACTTTGCTGGCCCC |
|  | Reverse | TTTTCGTTGGGTGAGGGGAG |
| HIF2α | Forward | CTGTATGGTCAGCTCAGCCC |
|  | Reverse | GGCTGTCAGACCCGAAAAGA |
| PGM5-AS1 | Forward | CAATGTTTTAGTTGAGACAGGAGGA |
|  | Reverse | AGATTCATCACCTCAAAACCTATCT |
| MFSD4A-AS1 | Forward | TGCCTGCATATCCTGTACCAT |
|  | Reverse | CCCAATAGGCATCTCTCTGGT |
| PRDM16-DT | Forward | TGCTCAGATTCGAACCCAGAT |
|  | Reverse | TCAAGGGAGTTCAGGTTCACA |
| TRHDE-AS1 | Forward | CAGCTTTCTCGAACCTTGGG |
|  | Reverse | GAACACTTCTGTCCACTGCG |
| LINC00887 | Forward | CACAGCAGCCTCCTCTTAAAC |
|  | Reverse | CTTTTCTCTCCCATGCTGAGC |
| LINC00634 | Forward | GGAGCCCTCATTTCTTCGTG |
|  | Reverse | CACGGTCTTATCCCTCTGCA |
| LINC00473tv1 | Forward | TCTGTACGCGCCCTTGATTA |
|  | Reverse | GTGTCCGCTTTGCATTCAGA |
| LINC00473tv2 | Forward | CTTTGCGCAGTTCTCCATGGAA |
|  | Reverse | AACGAGCACCAGAGAATACTAGT |
| GATA3-AS1 | Forward | CAAATCCACTCTCTGCTCGC |
|  | Reverse | TGTTGTTCCTTCACCGCATG |
| LINC00461 | Forward | ACAATGCAAATAGAGGCCTGT |
|  | Reverse | TGACCTGGTTTCTGTTTGTGT |
| 887L-P1  (887L) | Forward | GACTTACTCCAAGAGCCCCG |
|  | Reverse | AGGGAGCACATTCTGTTTCTCA |
| 887L-P2 | Forward | CCTGCTTGGCAGGTAACAGA |
|  | Reverse | GATGCCTCAGTCGAAGGGAG |
| 887L-P3 | Forward | TCCTGCTTGGCAGGTAACAG |
|  | Reverse | AACGATGCCTCAGTCGAAGG |
| 887S-P1  (887S) | Forward | CCTAGGGCCATGTGTTGTCA |
|  | Reverse | GAGGCTAGGCTTAGGGCTTC |
| 887S-P2 | Forward | AGCCCTAAGCCTAGCCTCTT |
|  | Reverse | TGCCCGGTGCTATTCTGATT |
| HRE1  (887S promoter) | Forward | TGAACGTGTGTCCTGTGAGC |
|  | Reverse | AATGGAGGTCACCCATCGAG |
| **Name** | **Primer sequence** | |
| HRE2  (887S promoter) | Forward | TCTGCGGGACTTACTCCAAG |
|  | Reverse | GCTAGAATCAGTACTCCCGCC |
| Ctrl region 1  (887S promoter) | Forward | GTTCCCATCTCACCCGCATC |
|  | Reverse | GGCTCTTGGAGTAAGTCCCG |
| Ctrl region 2  (887S promoter) | Forward | GAGACTGGTCCAAGGTCACTG |
|  | Reverse | GGACACACAATCCAGAGTCCA |
| CpG  (CA9 promoter) | Forward | CCAGGGAGAGCCTGCATAGT |
|  | Reverse | TTTTGCAGAGATGGAGCCAAAG |
| Ctrl region  (CA9 promoter) | Forward | CTTGCTTTTCATTCAAGCTCAAGT |
|  | Reverse | AGCCAGCCTCAGCTAACCC |
| HRE  (CA9 promoter) | Forward | GGCCTAAGCCCATTCTTGGT |
|  | Reverse | CTGGGCAAAGGGAAGGCATA |
| CA9 | Forward | AGAAATCGCTGAGGAAGGCT |
|  | Reverse | TCAGCTGTAGCCGAGAGTCA |
| CA2 | Forward | ACTGGGGTTCACTTGATGGAC |
|  | Reverse | GTTTAGCGCTGCCAACCTTC |
| CA12 | Forward | CCAAGTGCAAGTCTGTACTGC |
|  | Reverse | TGGGCCTCAGTCTCCATCTT |
| ATP13A3 | Forward | ATGGTTACAATTTGAGTCGCTGG |
|  | Reverse | AAAGAACGCGAATTTTTGCACA |
| CPN2 | Forward | GCATCTTCGACACCAACTACA |
|  | Reverse | CGCAGTAGGTCTGGATGTTCAG |
| HES1 | Forward | CTGAGCACAGACCCAAGTGT |
|  | Reverse | GAGTGCGCACCTCGGTATTA |
| LRRC15 | Forward | ATACATCCCTGACGGAGCCT |
|  | Reverse | CAAGCCCATCAAAAGTGCCC |
| OPA1 | Forward | ATCTGTGGATGCTGAACGCA |
|  | Reverse | GAATCCTGCTTGGACTGGCT |
| DNMT1 | Forward | TACCTGGACGACCCTGACCTC |
|  | Reverse | CGTTGGCATCAAAGATGGACA |
| ACTB | Forward | CATGTACGTTGCTATCCAGGC |
|  | Reverse | CTCCTTAATGTCACGCACGAT |
| Primer1 | Forward | GCCTTTGAGATTCCTGCGAA |
|  | Reverse | TGCCTCAGTCGAAGGGAGAT |
| Primer2 | Forward | TGCTTGGCAGGTTCCTATCT |
|  | Reverse | CGCACAAGGTTTTCCCCATC |
| Primer3 | Forward | AGAATCCAAGGACTTGTGCT |
|  | Reverse | GCCTTTGAGATTCCTGCGAA |
| Primer4 | Forward | AGAAAGGGGTGATGTTGGCA |
|  | Reverse | CCCTTGCTGGGGAATGTGAA |
| Primer5 | Forward | GATTCCTCTCCCATCTTCCACCC |
|  | Reverse | TCGCAGGAATCTCAAAGGCC |
| **Name** | **Primer sequence** | |
| Primer6 | Forward | TGTGGACTTGGCAGTTTGGA |
|  | Reverse | AAGCAACGCAGAGCATTTGG |
| Primer7 | Forward | CTCCCCAGCCATCCTACTCT |
|  | Reverse | ATACAAGCCAGCCCGAGAAG |
| Primer8 | Forward | AGAAAGGGGTGATGTTGGCA |
|  | Reverse | CCCTTGCTGGGGAATGTGAA |
| Primer9 | Forward | TCTTCAGATCAGGACGCACG |
|  | Reverse | AAGCCAGCCCGAGAAGAATG |

**Supplementary Table3: siRNA, ASO, ChIRP, FISH and Northern blot probe sequences**

| **Target** | **Tool** | **Target sequence** |
| --- | --- | --- |
| CA9 | siRNA-siCA9 | GTTGCTGTCTCGCTTGGAA |
| DNMT1 | siRNA-siDNMT1-1 | CAATGAGACTGACATCAAA |
|  | siRNA-siDNMT1-2 | GAGGCCTATAATGCAAAGA |
| HIF1α | siRNA-siHIF1α | GTAGCCTCTTTGACAAACT |
| HIF2α | siRNA-siHIF2α | CGCTCAGCCTATGAATTCT |
| 887S | ASO | TCCCATTTCAGGCAGGACTT |
| 887S | ChIRP-Probe-1 | GTGTCTGAGCAGAAAATGAA-/3bio/ |
|  | ChIRP-Probe-2 | CCAGTAATATAACCAGGCAC-/3bio/ |
|  | ChIRP-Probe-3 | CTTCAGACAATTTCAGCCTC-/3bio/ |
|  | ChIRP-Probe-4 | GACAGTCTTCCCTCCAGATC-/3bio/ |
|  | ChIRP-Probe-5 | CTCCGATAAACAGGTGAAAC-/3bio/ |
|  | ChIRP-Probe-6 | TCAGATGGTCCTTCAACTTC-/3bio/ |
|  | ChIRP-Probe-7 | CTCAGCCAGAATCCTAAACC-/3bio/ |
|  | ChIRP-Probe-8 | TTGTTTGGAATCTTCTGGTC-/3bio/ |
|  | ChIRP-Probe-9 | CATAGGCACCATCACAAT-/3bio/ |
|  | ChIRP-Probe-10 | TAGAAGAGGCTAGGCTTAGG-/3bio/ |
|  | ChIRP-Probe-11 | CGGTGCTATTCTGATTCATA-/3bio/ |
| 887L | ChIRP-Probe-1 | AAGCAGCATCATTGGAGATC-/3bio/ |
|  | ChIRP-Probe-2 | AGGAAGAGTGAATGGAGGTC-/3bio/ |
|  | ChIRP-Probe-3 | AGTCCTTGGATTCTTTGTCA-/3bio/ |
|  | ChIRP-Probe-4 | ACTCCGATAAACAGGTGAAA-/3bio/ |
|  | ChIRP-Probe-5 | CAGGAACCTTGTCTCTTGTT-/3bio/ |
|  | ChIRP-Probe-6 | GTGAGCATCTCTTCTGATTG-/3bio/ |
|  | ChIRP-Probe-7 | GAGATGATGAGTCCAGACAA-/3bio/ |
|  | ChIRP-Probe-8 | AGATGAGGACCAAGAAATGT-/3bio/ |
|  | ChIRP-Probe-9 | TGGATTCTCAGCATACACAA-/3bio/ |
|  | ChIRP-Probe-10 | ATTGGCTGAGATGGGAAATC-/3bio/ |
|  | ChIRP-Probe-11 | AATAGTGGTGACGAGAAGTG-/3bio/ |
|  | ChIRP-Probe-12 | TTGGAATGAATGTGGCTGAC-/3bio/ |
|  | ChIRP-Probe-13 | AAGAGTTCCAAGCAGAAGAA-/3bio/ |
|  | ChIRP-Probe-14 | AATTCTCTACAGATGACGCA-/3bio/ |
|  | ChIRP-Probe-15 | AAGATAGAGGAGAATCGGTG-/3bio/ |
|  | ChIRP-Probe-16 | AGGACAGGCAATATAGACAC-/3bio/ |
|  | ChIRP-Probe-17 | GGTAAATTCTGGTGAACGAC-/3bio/ |
|  | ChIRP-Probe-18 | TGATTAAAAGTGACAGCCAG-/3bio/ |
|  | ChIRP-Probe-19 | ACACTTCAAGCAAGAGGATA-/3bio/ |
|  | ChIRP-Probe-20 | CATCACCACAATACCTCTCA-/3bio/ |
|  | ChIRP-Probe-21 | TTACAGGCTCTCAACATTGA-/3bio/ |
| **Target** | **Tool** | **Target sequence** |
| ACTB(β-actin) | FISH-probe (5’ Cy5/Cy3) | CCTCCTGAGCGCAAGTACTCCGTGT |
| NC | FISH-probe (5’ Cy5/Cy3) | GTGTAACACGTCTATACGCCCA |
| 887S | FISH-probe (5’ Cy3)-1 | GAAGTGCCGGTTTAAAAGTGA |
|  | FISH-probe (5’ Cy3)-2 | ATAGGCACCATCACAATTCAAC |
|  | FISH-probe (5’ Cy3)-3 | CTTTCTCTAATTGAAGACCAGG |
| 887L | FISH-probe (5’ Cy5)-1 | CGAGGGGAGTGTATAAGTCATC |
|  | FISH-probe (5’ Cy5)-2 | AAAGAGTGACAGGTCAAAGGGA |
|  | FISH-probe (5’ Cy5)-3 | AATACCTCTCATACTCTTTTGA |
| 887S | Northern blot-probe | /5bio/-TAGCCCAACTCTCTTCAACAC  CAAACCCCTTATGCATCTGTCAG  ATGGTCCTTCAACTTC |
| 887L | Northern blot-probe | /5bio/-AGTCCGTGTAAGGCAAGCGG  GACTGTAAGCTCCATGAGGACAGG  AACCTTGTCTCTTGTT |
