# Supplementary material for: A pair of long intergenic non-coding RNA LINC00887 variants act antagonistically to control Carbonic Anhydrase IX transcription upon hypoxia in tongue squamous carcinoma progression
Source: BMC Biol. 2021 Sep 7;19:192. doi: 10.1186/s12915-021-01112-2 (PMC8422755; doi:10.1186/s12915-021-01112-2)
Supplement: Supplementary file 2 — Additional File 2: Fig S1. Up-regulation of LINC00887 is correlated with poor survival rate of TSCC patients. Fig S2. The variety, existence and relative abundance of 887S, 887L and other LINC00887 variants. Fig S3. The expression pattern of 887S and 887L in the indicated cell lines. Fig S4. 887S and 887L subcellular localization and coding potential. Fig S5. Efficiency of gain-of-function or loss-of-function experiments in the indicated 887S and 887L knockdown or overexpression cells. Fig S6. CA9 promotes tumor progression in TSCC. Fig S7. Expression level of CA9 in the indicated 887S or 887L modulated cells. Fig S8. 887S and 887L regulates intracellular pH in TSCC. Fig S9. Effects of 887S and 887L on xenograft growth in Balb/c (nu/nu) mice. Fig S10. Effects of ASO-mediated 887S knockdown. Fig S11. In vitro transcribed 887S visualized by agarose gel with 1% formaldehyde. Fig S12. 887L promotes TSCC progression under normoxia. [file 12915_2021_1112_MOESM2_ESM.docx]

**Supplementary Figures**


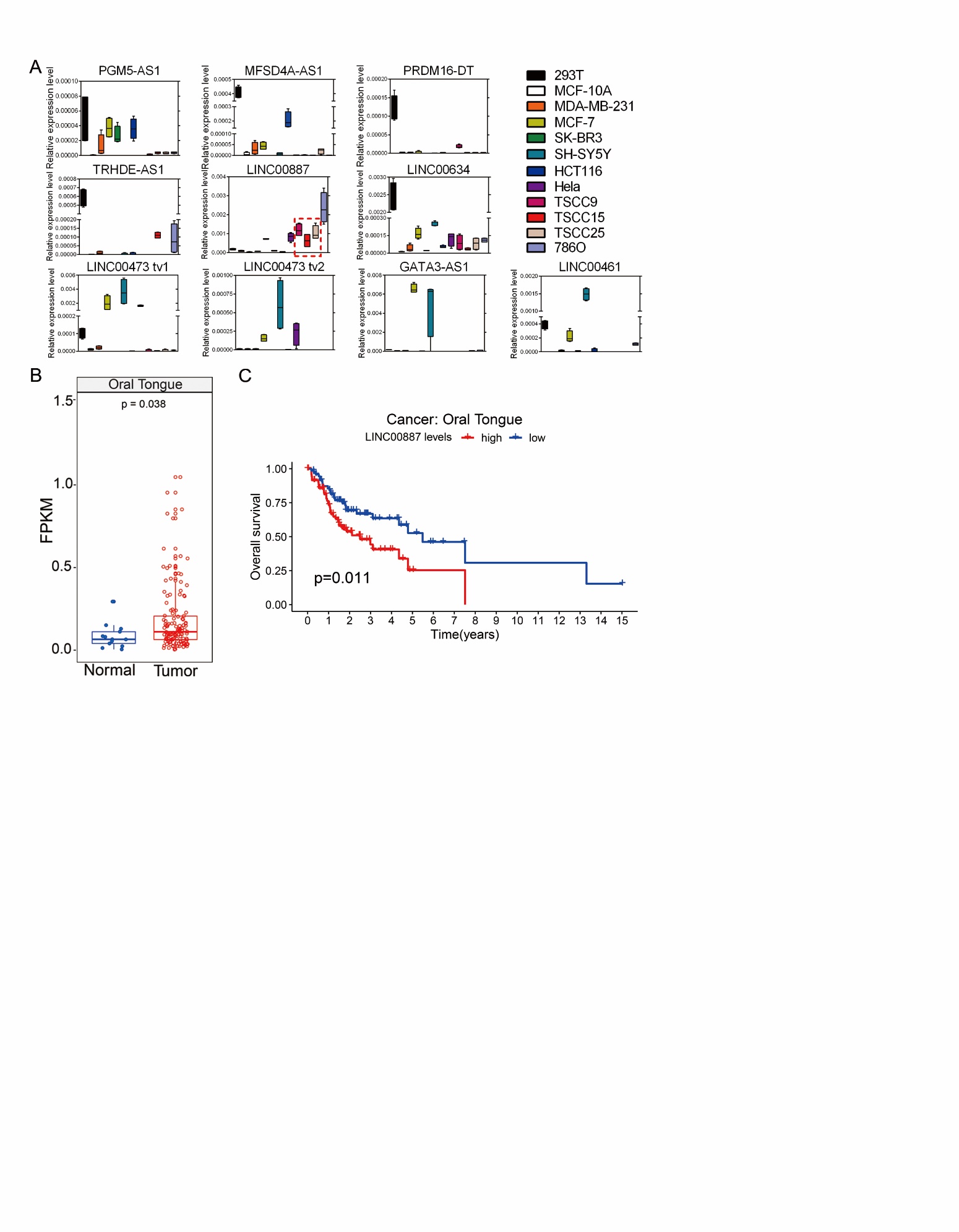


**Fig S1. Up-regulation of *LINC00887* is correlated with poor survival rate of TSCC patients.** (A) The expression levels of top 10 CVAA lncRNAs in the indicated normal and tumor cell lines detected by RT-qPCR (n=4). (B) The expression levels of *LINC00887* in oral tongue normal or tumor samples retrieved from TCGA. Normal, n=15; Tumor, n=147. (C) Survival analysis of *LINC00887* in TCGA retrieved TSCC patients. n (high groups) =73, n (low groups) =74. Data are shown as means ± SEMs. P values are calculated using Student’s t test.


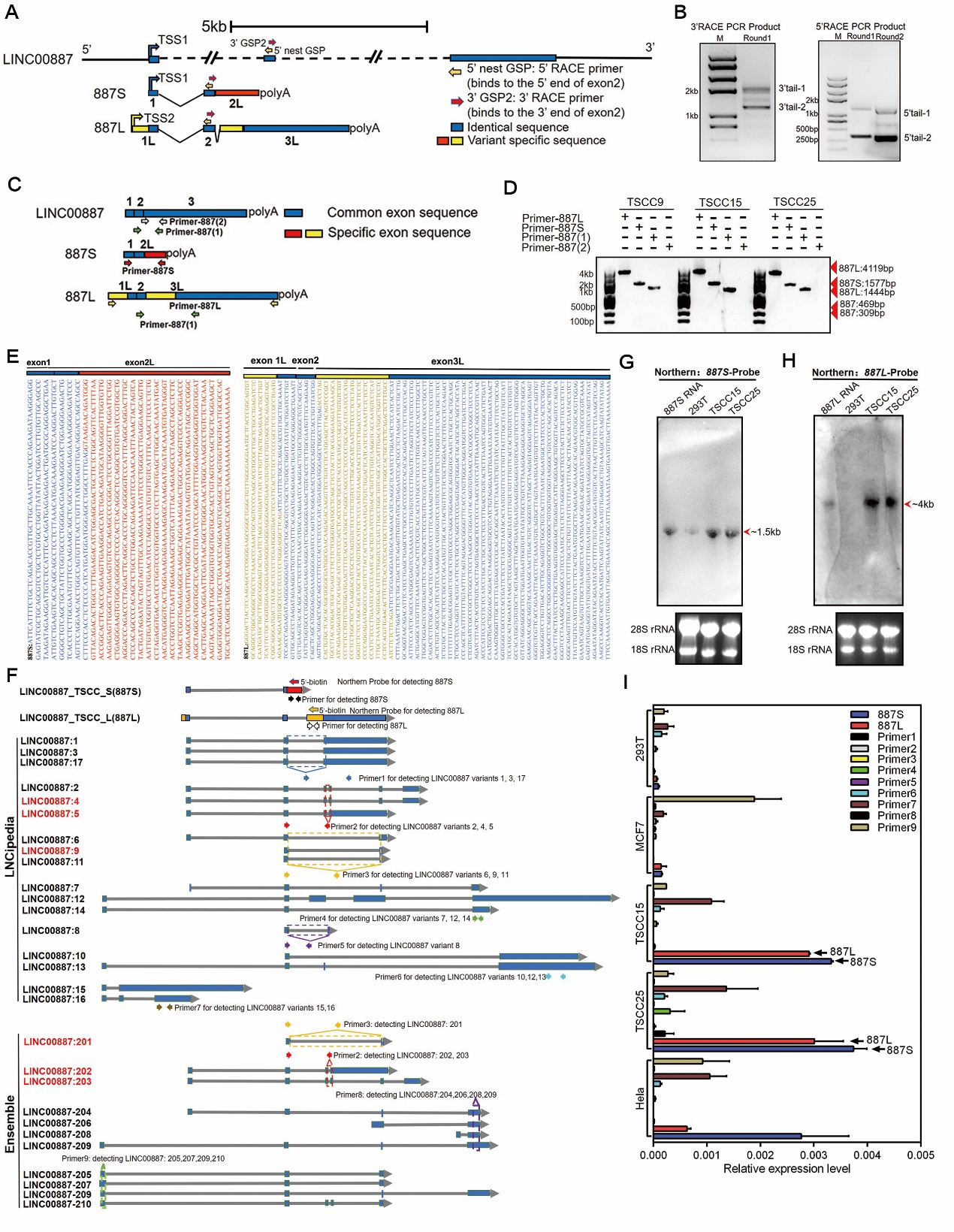


**Fig S2. The variety, existence and relative abundance of *887S*, *887L* and other *LINC00887* variants.** (A) A schematic illustration for the position targeted by RACE primers for *887S* and *887L*. (B) Electrophoresis gel images of 3’RACE and 5’RACE showed the identification of two major products of *LINC00887*. (C, D) A schematic view of the primer designed strategy (C) and the electrophoresis gel image of RT-PCR (D) for detecting *LINC00887*, *887S*, and *887L*. (E) Sequence of *887S* (left panel) and *887L* (right panel). (F) A schematic view of *887S*, *887L* and *LINC00887* variants shown in LNCipedia and Ensemble database*.* Overlapped *LINC00887* variants in two databases are marked in red. The primers and probes used for RT-qPCR assay and Northern blot assay are also indicated. (G, H) Image of Northern blot for *887S* (G) and *887L* (H). *In vitro* transcript *887S* (G) and *887L* (H) RNA were respectively used as positive controls and position markers. (I) The expression levels of *887S*, *887L*, and *LINC00887* variants illustrated in (F) detected by RT-qPCR in the indicated cell lines (n=3). Data are shown as means ± SEMs. P values are calculated using Student’s t test.


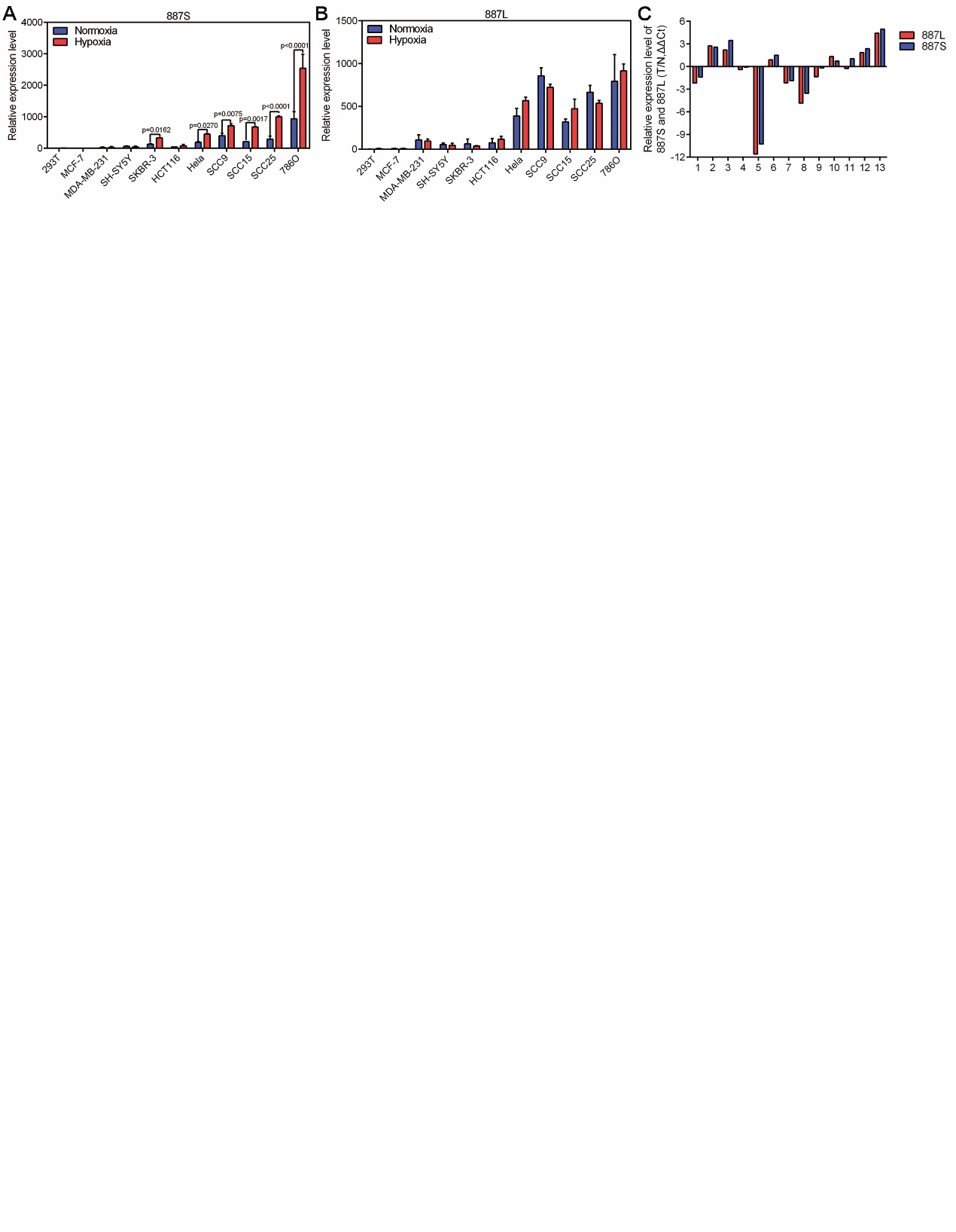


**Fig S3. The expression pattern of *887S* and *887L* in the indicated cell lines.** (A, B) Relative expression levels of *887S* (A) and *887L* (B) in the indicated cells under both normoxic and hypoxic conditions (n=3). (C) Relative expression levels of *887S* and *887L* in the in-house collected TSCC and paraneoplastic samples (n=13). Data are shown as means ± SEMs. P values are calculated using Student’s t test.


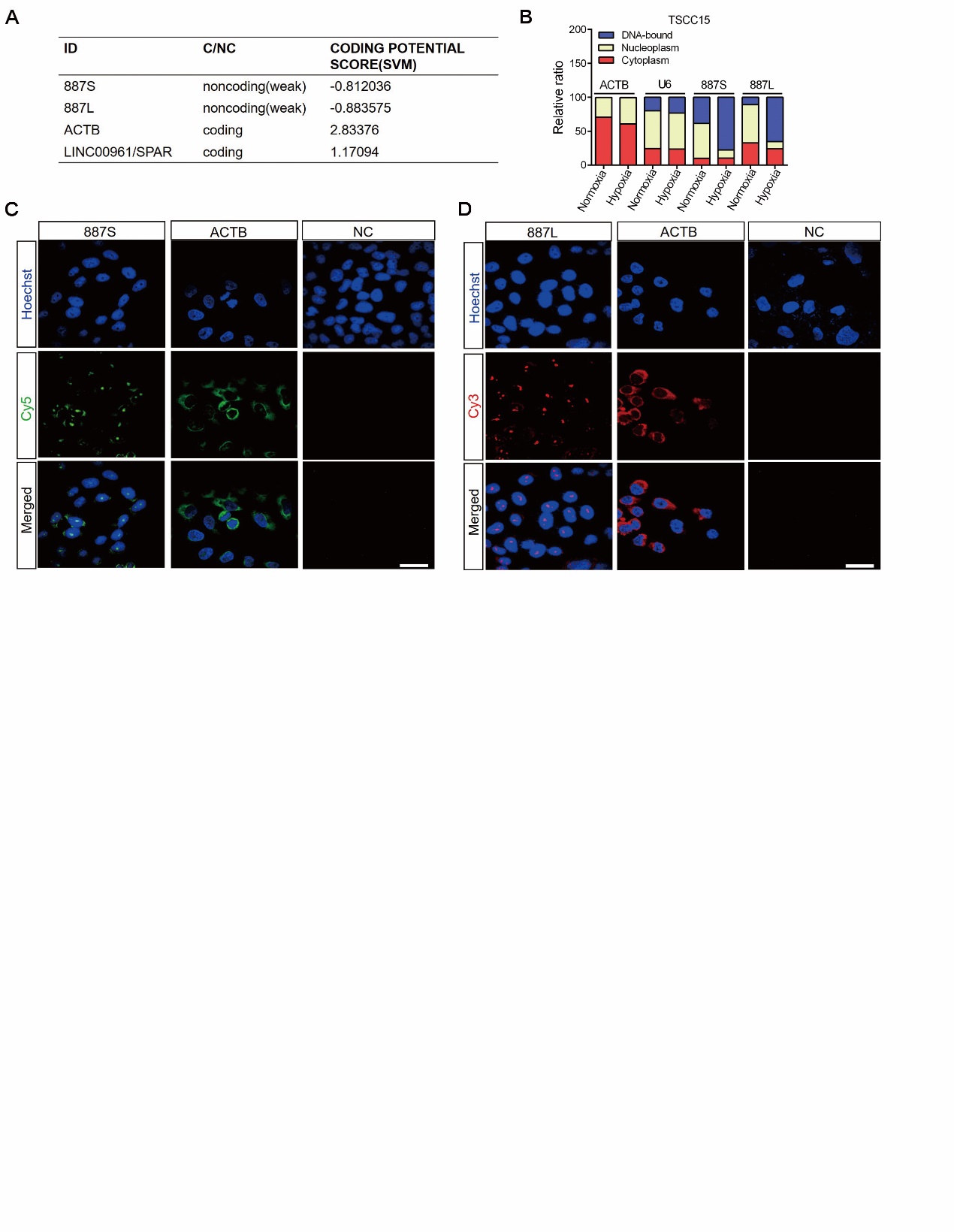


**Fig S4. *887S* and *887L* subcellular localization and coding potential.** (A) Protein-coding potential scores of the indicated RNAs analyzed by CPC (coding potential calculator, http://cpc.cbi.pku.edu.cn). ACTB: β-actin serving as a control protein coding gene; LINC00961/SPAR: a control for lncRNA gene which has the ability to derive micropeptide (88). (B) The subcellular distributions of *887S*, *887L* determined by fractionationing assay in the presence of normoxia or hypoxia (n=2). ACTB and U6 were used as cytoplasmic and nuclear marker, respectively. (C, D) RNA-FISH showed the subcellular localization of *887S* (C) and *887L* (D). ACTB and U6 serve as markers for cytoplasm-expressing RNA and nuclear-expressing RNA, respectively. Scale bar, 20 mm.


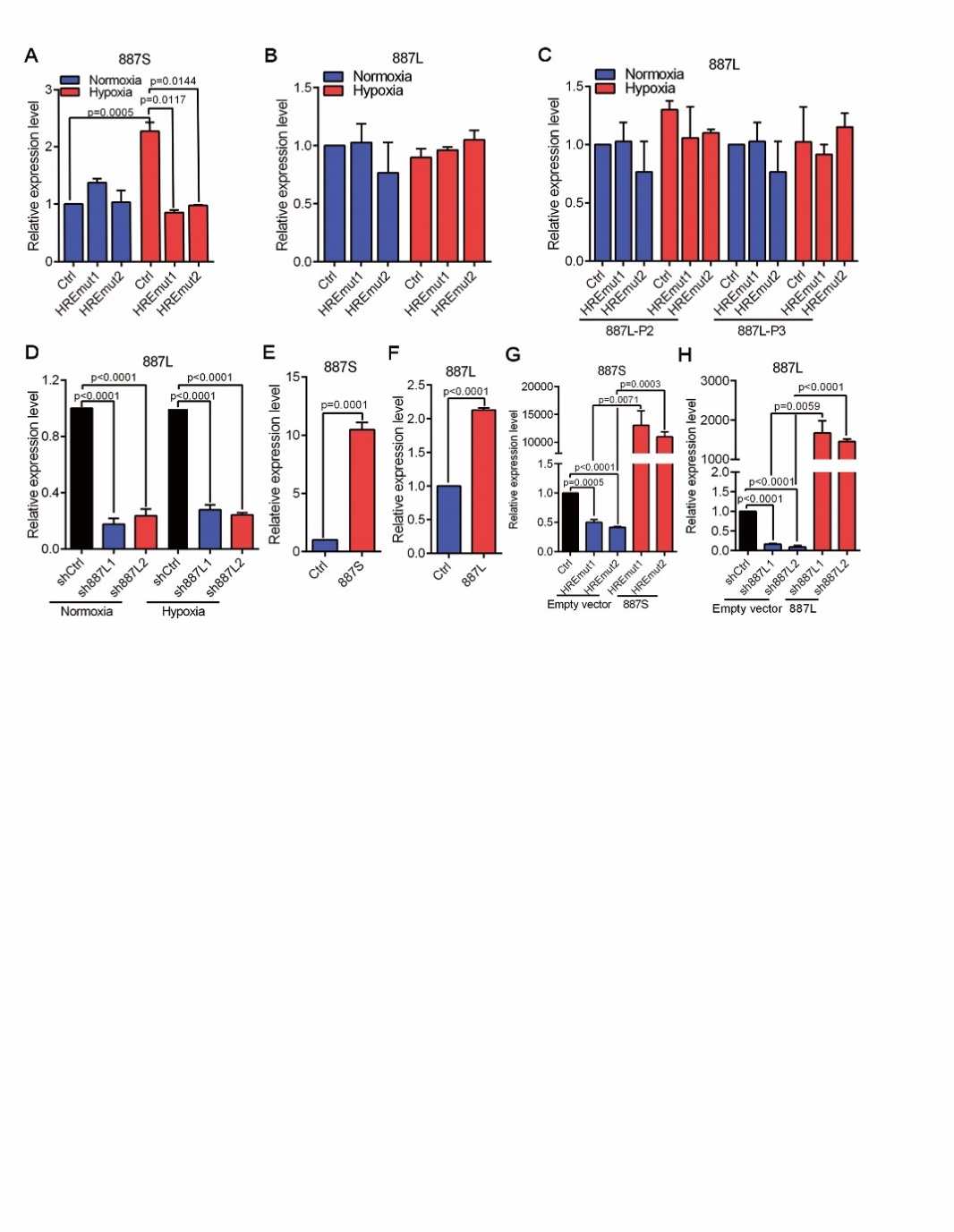


**Fig S5. Efficiency of gain-of-function or loss-of-function experiments in the indicated *887S* and *887L* knockdown or overexpression cells.** (A) Relative expression levels of *887S* in the indicated HRE mutant cell lines under hypoxia (n=3). (B, C) Relative expression levels of *887L* in the indicated HRE mutant cell lines under normoxia and hypoxia (n=3). (D) Knockdown efficiency of *887L* in the indicated shRNA-mediated TSCC15 stable cells under normoxia and hypoxia (n=3). (E, F) Overexpression efficiency of *887S* (E) and *887L* (F) (n=3). Related to Figure 3E. (G) Overexpression efficiency of *887S* in the indicated HRE mutant cells (n=3). Related to Figure 4A, B and E, F. (H) Overexpression efficiency of *887L* in the indicated *887L* knockdown cells (n=3). Related to Figure 4C, D and G, H. Data are shown as means ± SEMs. P values are calculated using Student’s t test.


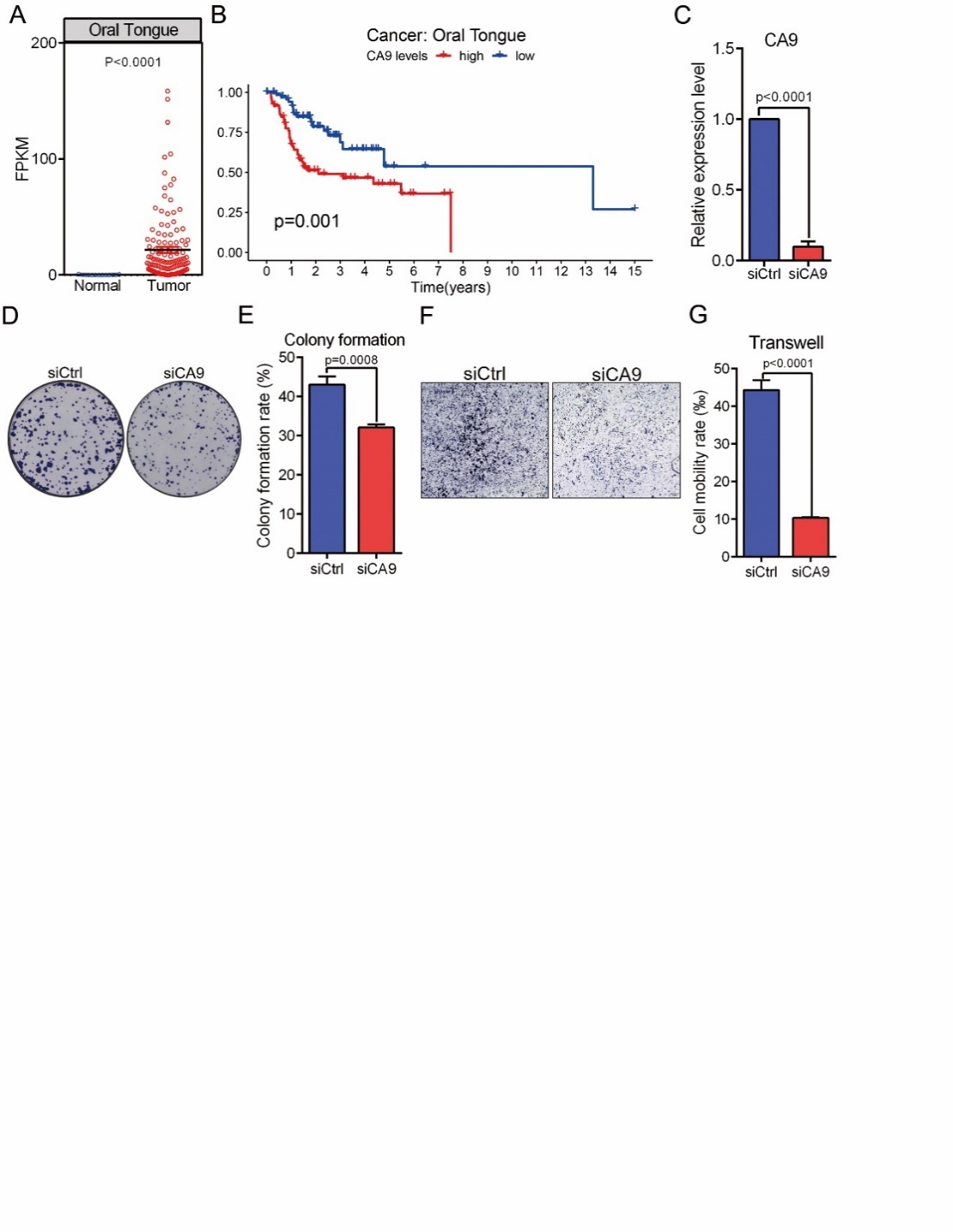


**Fig S6. CA9 promotes tumor progression in TSCC.** (A) The expression levels of CA9 in TCGA retrieved oral tongue normal and tumor specimens. Normal, n=15; Tumor, n=147. (B) Survival analysis of CA9 in TCGA retrieved TSCC patients. n (high groups) =73, n (low groups) =74. (C) The efficiency of CA9 siRNA (n=3). (D-G) Representative images (D, F) and the according statistical analysis of colony formation (E, n=3) and transwell (G, n=3) assays in CA9 knockdown TSCC cells under nomorxia. Data are shown as means ± SEMs. P values are calculated using Student’s t test.


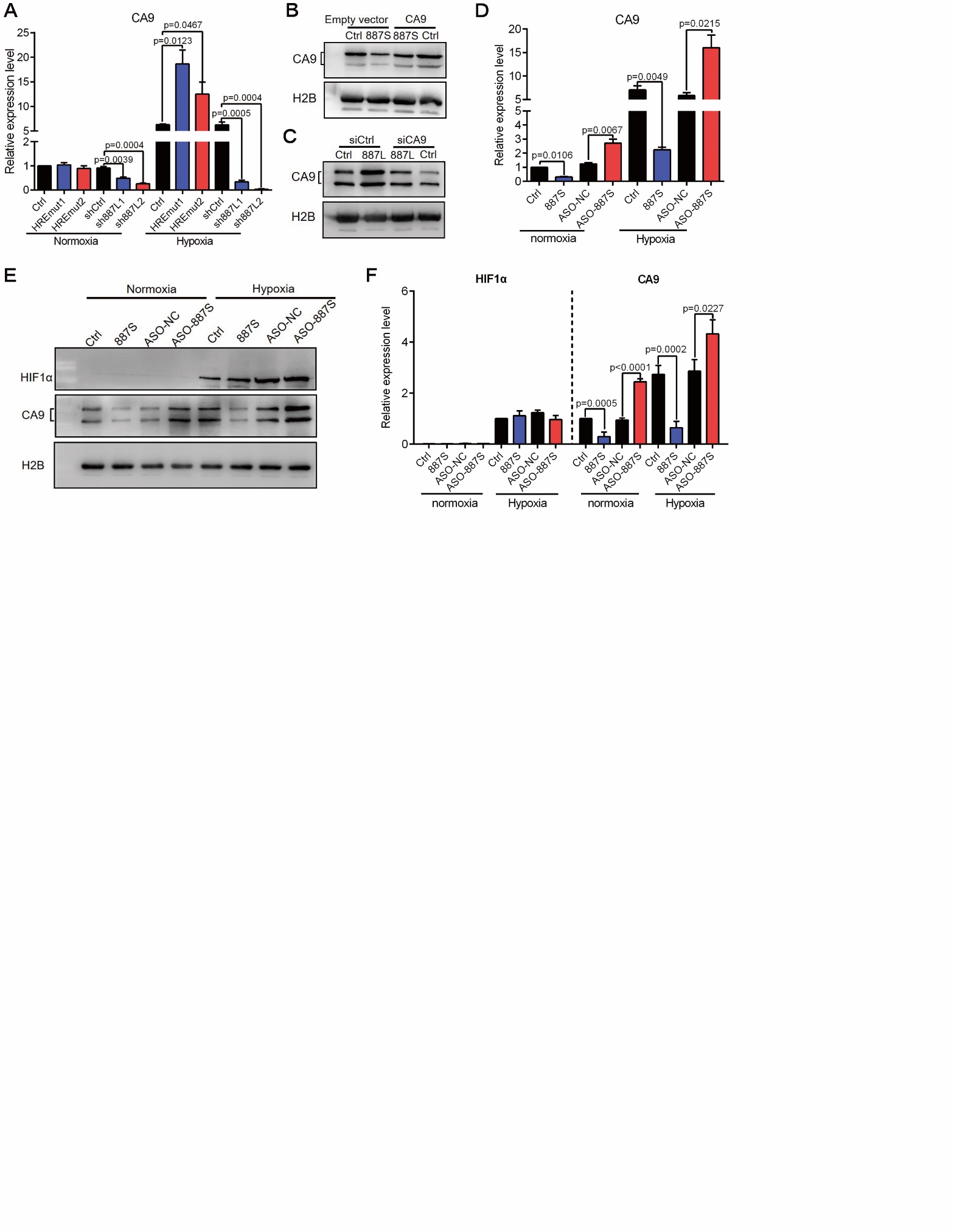


**Fig S7. Expression level of CA9 in the indicated *887S* or *887L* modulated cells.** (A) Relative expression levels of CA9 in *887S* knockout cells or *887L* knockdown cells under normoxic or hypoxic condition (n=3). (B) The expression levels of CA9 protein under the indicated treatments (n=3). Related to Figure 4L, M. (C) The expression levels of CA9 protein under the indicated treatments (n=3). Related to Figure 4N, O. (D, E) Relative mRNA expression level (D) or protein level (E) of CA9 in *887S* knockdown or overexpression cells under normoxic or hypoxic condition (n=3). (F) Quantitation of the relative protein level changes of HIF1α and CA9 (n=3). Data are shown as means ± SEMs. P values are calculated using Student’s t test.


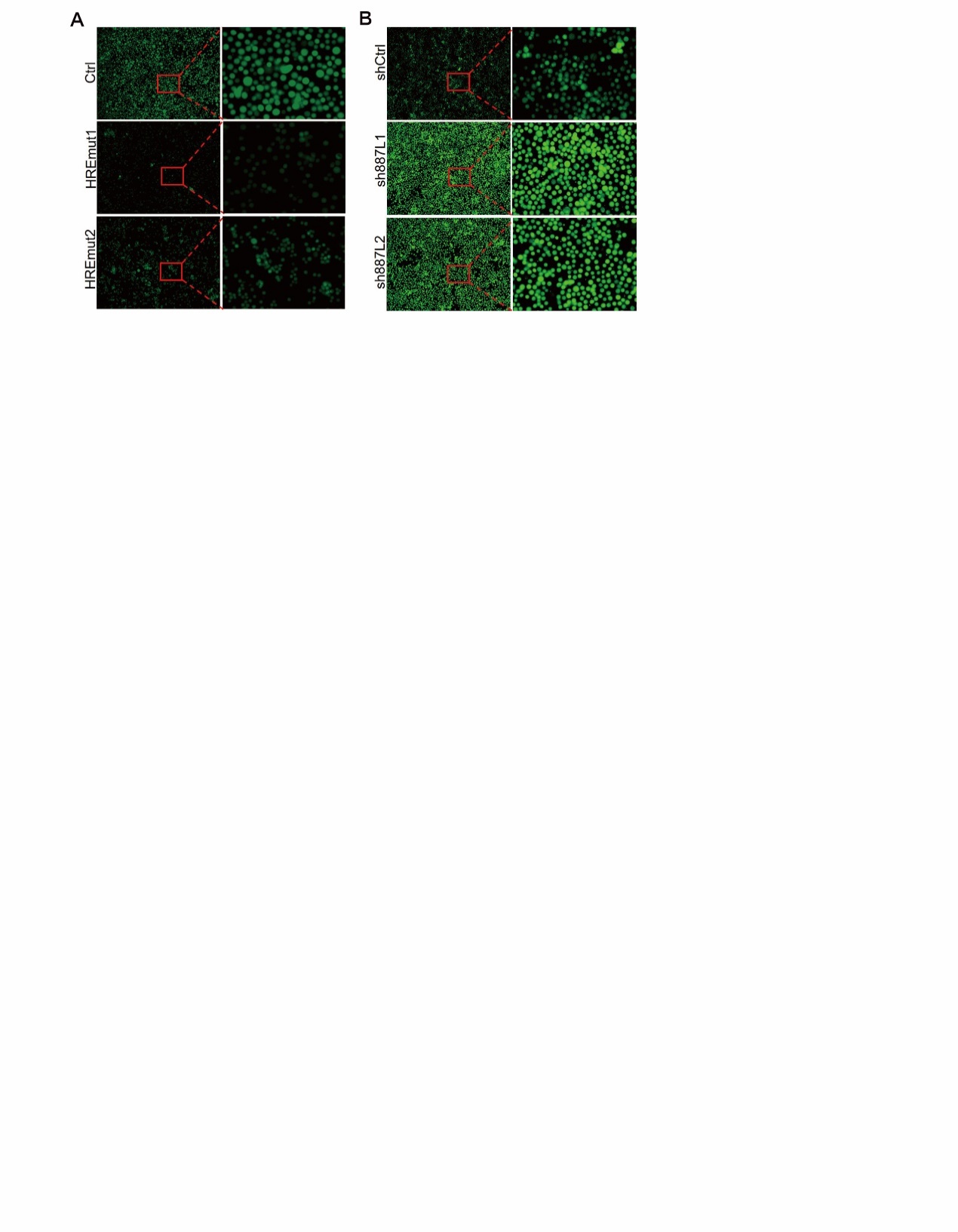


**Fig S8. *887S* and *887L* regulates intracellular pH in TSCC.** (A) Images of the fluorescence intensity detected by BCECF-AM assay in the indicated *887S* knockout cells in the presence of hypoxia. (B) Images of the fluorescence intensity detected by BCECF-AM assay in the indicated *887L* knockdown cells in the presence of normoxia.


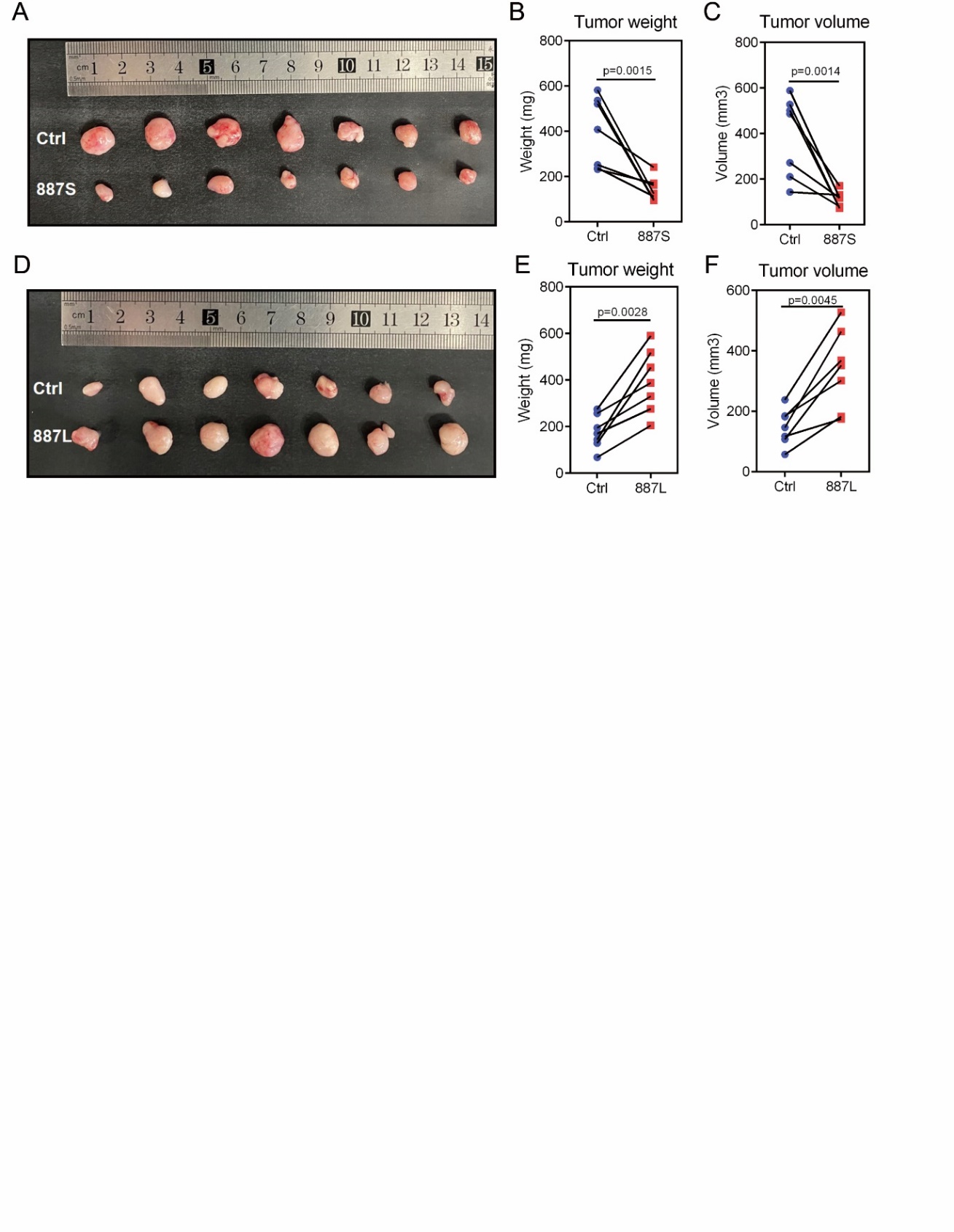


**Fig S9. Effects of *887S* and *887L* on xenograft growth in Balb/c (nu/nu) mice.** (A-C) Effects of *887S* overexpression in TSCC25 cells on tumor weights and tumor volumes in the subcutaneous xenografts mice models. (D-F) Effects of *887L* overexpression in TSCC25 cells on tumor weights and tumor volumes in the subcutaneous xenografts mice models. P values are calculated using Student’s t test.


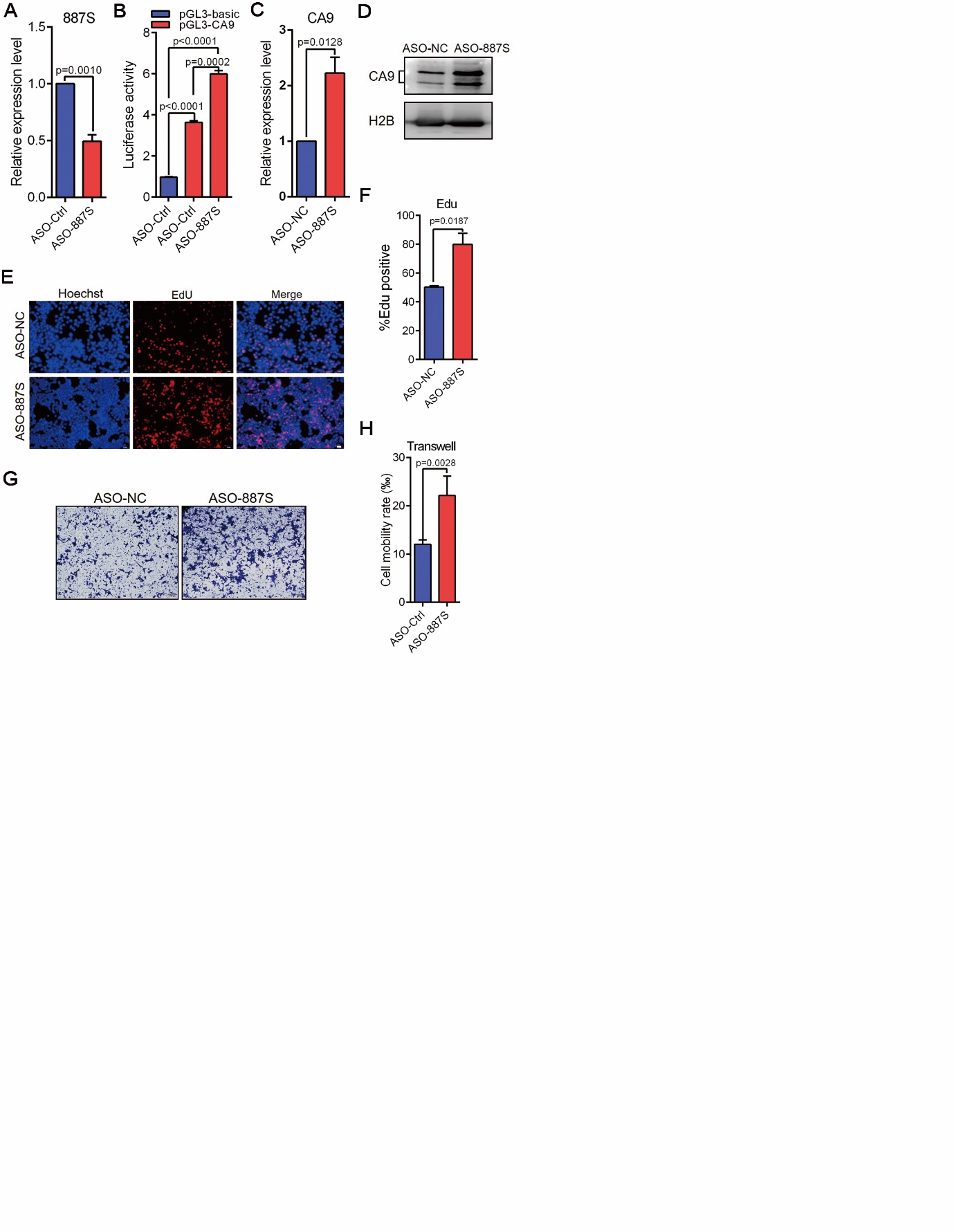


**Fig S10. Effects of ASO-mediated *887S* knockdown.** (A) Knockdown efficiency of *887S* ASO (n=3). (B) Activity change of *CA9* promoter in the indicated ASO-mediated *887S* knockdown cells. (C, D) The RNA (C) or protein (D) expression level of CA9 in the indicated ASO-mediated *887S* knockdown cells. (E, F) Representative images (E) and the according statistical analysis (F, n=3) of Edu incubation assay in the indicated ASO-mediated *887S* knockdown cells. Scale bar, 1mm. (G, H) Representative images (G) and the statistical analysis (H, n=3) of transwell assay in the indicated ASO-mediated *887S* knockdown cells. Data are shown as means ± SEMs. P values are calculated using Student’s t test.


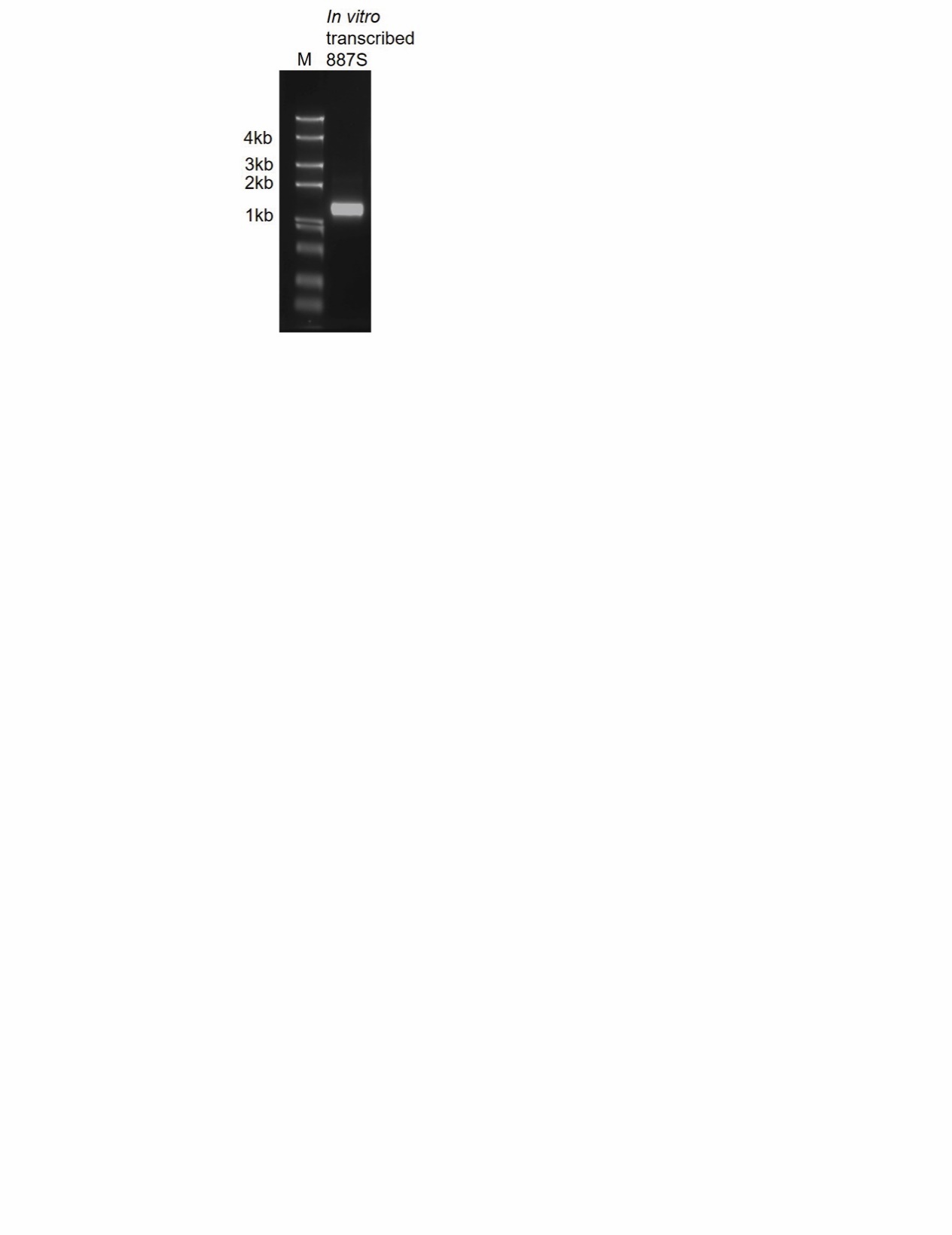


**Fig S11.** ***In vitro* transcribed *887S* visualized by agarose gel with 1% formaldehyde.** Related to Figure 6A.


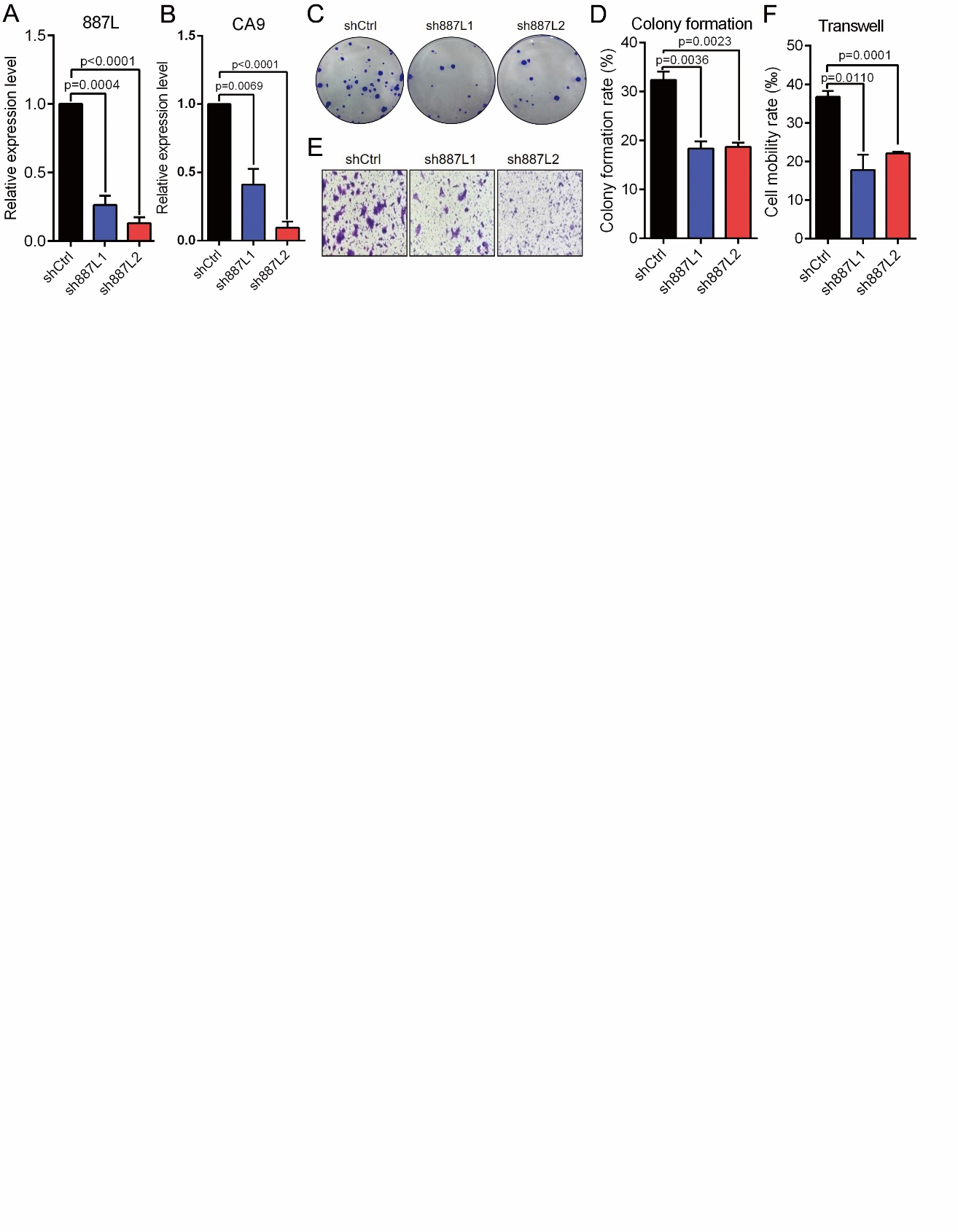


**Fig S12. *887L* promotes TSCC progression under normoxia.** (A, B) Relative expression level of *887L* (A) or CA9 (B) in sh*887L*1 and sh*887L*2 cells upon normoxia (n=3). (C-F) Representative images (C, E) and the according statistical analysis of colony formation (D, n=3) and transwell (F, n=3) assays in *887L* knockdown TSCC cells. Data are shown as means ± SEMs. P values are calculated using Student’s t test.
