# Supplementary material for: A pair of long intergenic non-coding RNA LINC00887 variants act antagonistically to control Carbonic Anhydrase IX transcription upon hypoxia in tongue squamous carcinoma progression
Source: BMC Biol. 2021 Sep 7;19:192. doi: 10.1186/s12915-021-01112-2 (PMC8422755; doi:10.1186/s12915-021-01112-2)

Fig 2D upper Panel

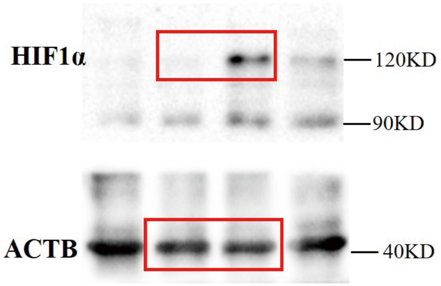

Fig 2D lower panel

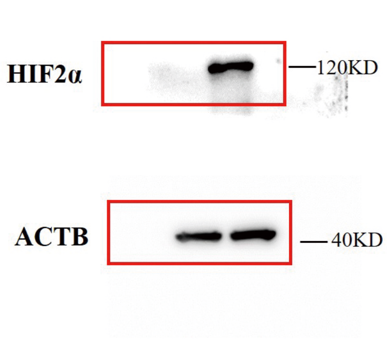

Fig 2F upper paneL

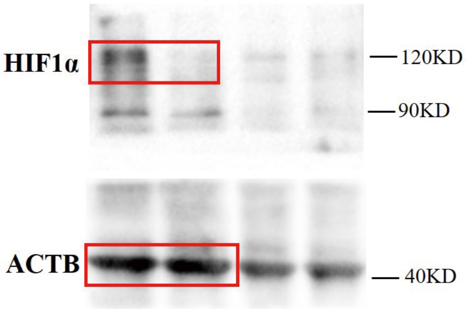

Fig 2F lower panel

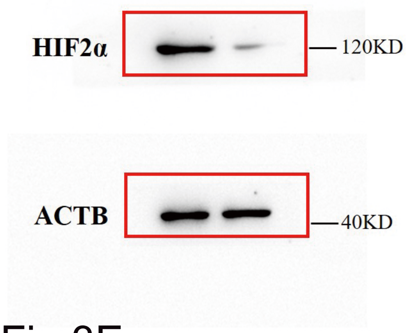

Fig 2J

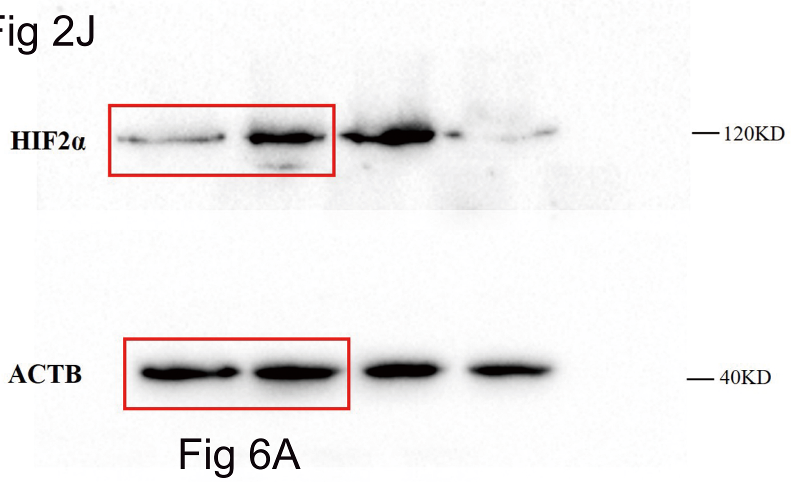

Fig 3E

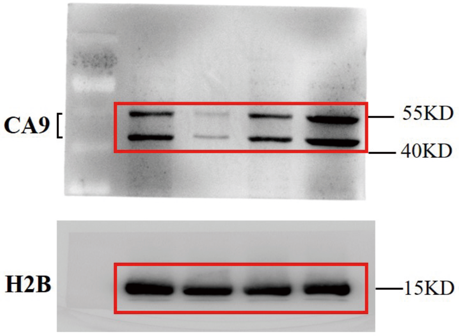

Fig 6A

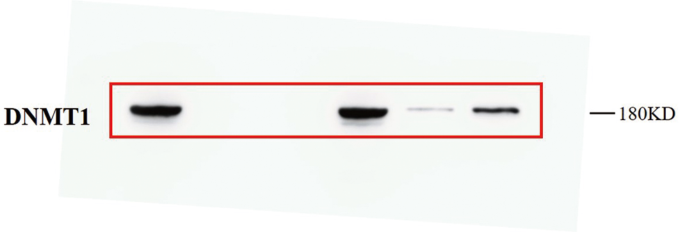

Fig 6D

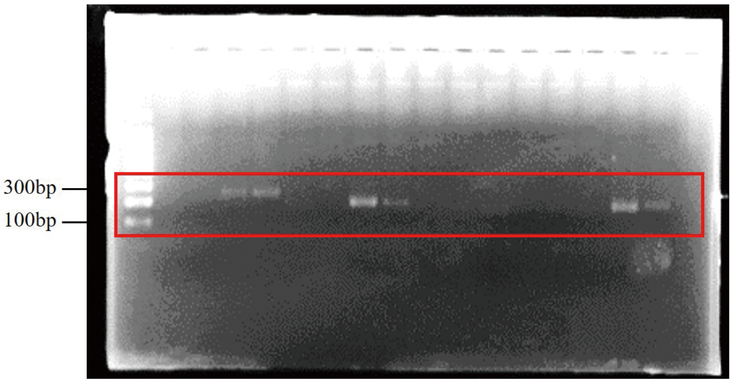

Fig 6N

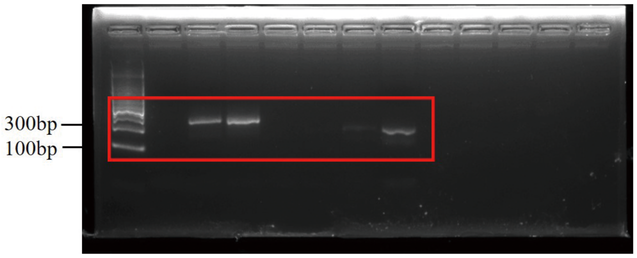

Fig 7E

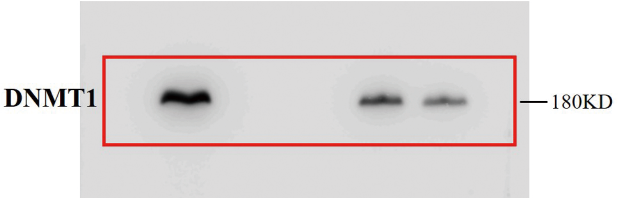

Fig 7G

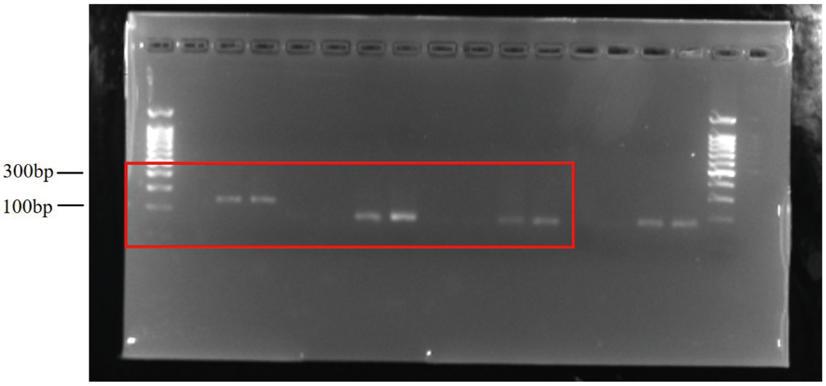

Fig S2B

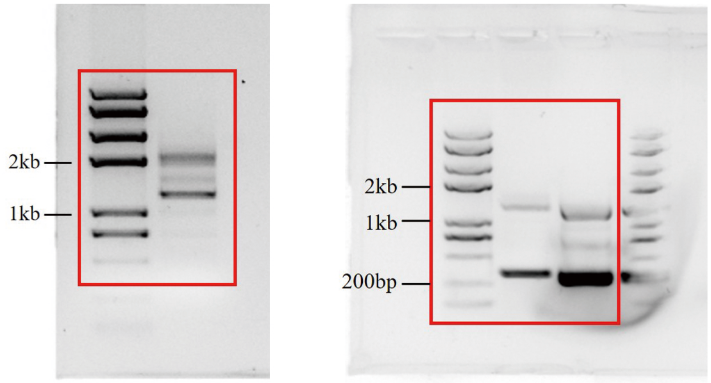

Fig S2D

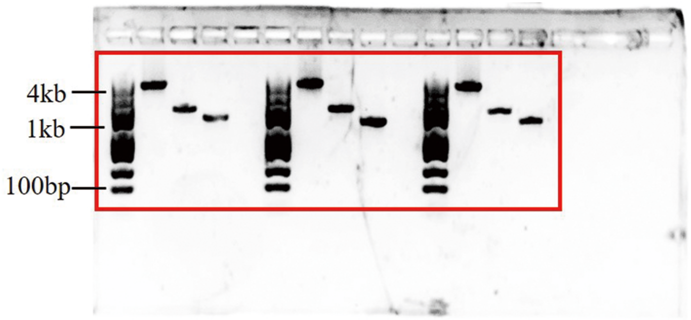

Fig S2G

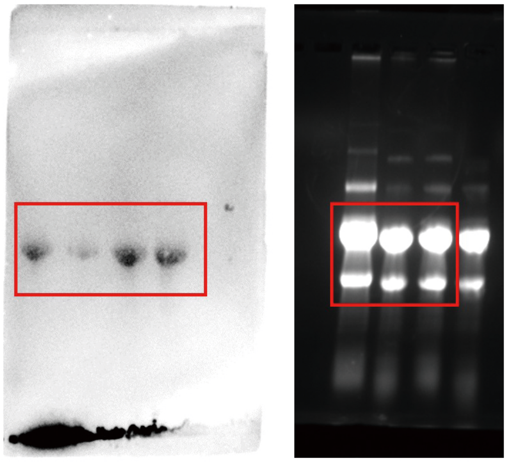

Fig S2H

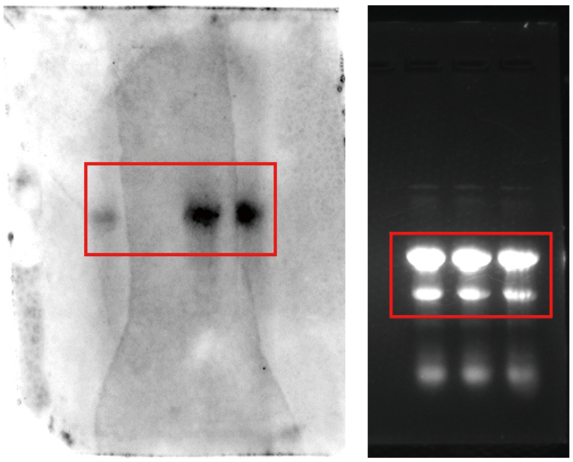

Fig S7B

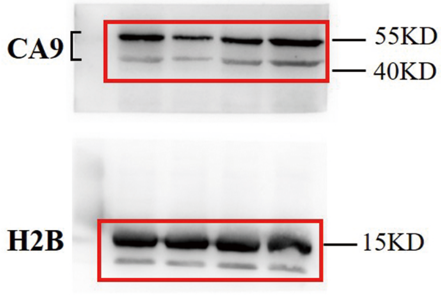

Fig S7C

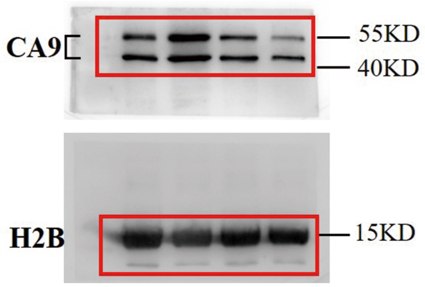

Fig S11

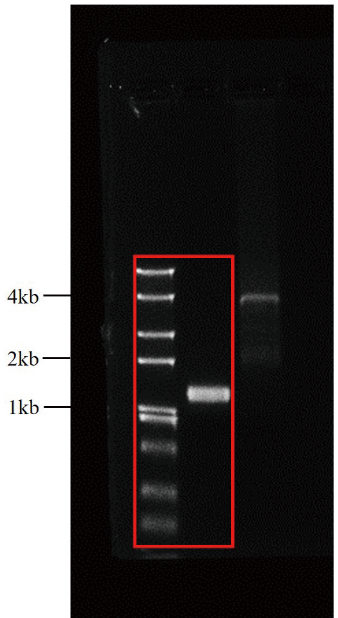

Fig S7E

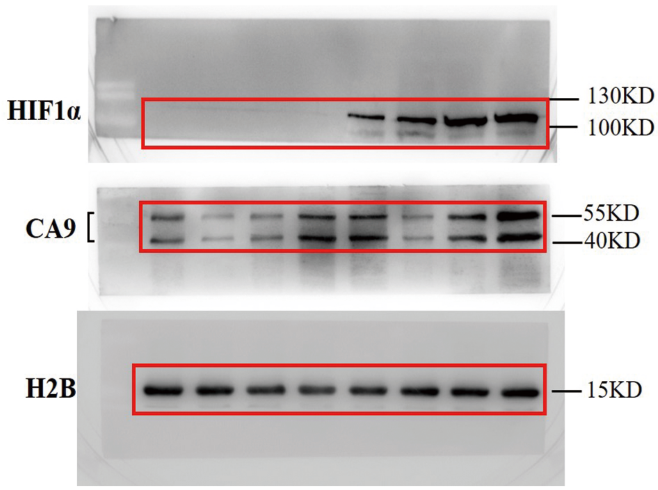

Fig S10D

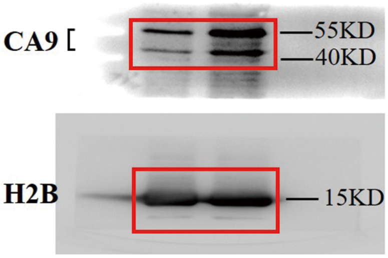

Supplement: Supplementary file 3 — Additional File 3. Raw blots and gel datasets [file 12915_2021_1112_MOESM3_ESM.pdf]
